# Genome-Wide Analysis of Heat Shock Protein Family and Identification of Their Functions in Rice Quality and Yield

**DOI:** 10.3390/ijms252211931

**Published:** 2024-11-06

**Authors:** Hong Wang, Sidra Charagh, Nannan Dong, Feifei Lu, Yixin Wang, Ruijie Cao, Liuyang Ma, Shiwen Wang, Guiai Jiao, Lihong Xie, Gaoneng Shao, Zhonghua Sheng, Shikai Hu, Fengli Zhao, Shaoqing Tang, Long Chen, Peisong Hu, Xiangjin Wei

**Affiliations:** State Key Laboratory of Rice Biology, China National Center for Rice Improvement, China National Rice Research Institute, Hangzhou 311400, China; benxiwanghong@163.com (H.W.); sidracharagh005@gmail.com (S.C.); 13126531292@163.com (N.D.); lufeifei0506@163.com (F.L.); 13835410849@163.com (Y.W.); caoruijie@caas.cn (R.C.); maliuyang2018@163.com (L.M.); 15309894079@163.com (S.W.); jiaoguiai@caas.cn (G.J.); xielihong@caas.cn (L.X.); shaogaoneng@caas.cn (G.S.); shengzhonghua@caas.cn (Z.S.); hushikai@caas.cn (S.H.); zhaofengli@caas.cn (F.Z.); tangshaoqing@caas.cn (S.T.); chenlong@caas.cn (L.C.)

**Keywords:** heat shock protein, gene family, heat resistance, rice quality and yield

## Abstract

Heat shock proteins (Hsps), acting as molecular chaperones, play a pivotal role in plant responses to environmental stress. In this study, we found a total of 192 genes encoding Hsps, which are distributed across all 12 chromosomes, with higher concentrations on chromosomes 1, 2, 3, and 5. These Hsps can be divided into six subfamilies (sHsp, Hsp40, Hsp60, Hsp70, Hsp90, and Hsp100) based on molecular weight and homology. Expression pattern data indicated that these *Hsp* genes can be categorized into three groups: generally high expression in almost all tissues, high tissue-specific expression, and low expression in all tissues. Further analysis of 15 representative genes found that the expression of 14 *Hsp* genes was upregulated by high temperatures. Subcellular localization analysis revealed seven proteins localized to the endoplasmic reticulum, while others localized to the mitochondria, chloroplasts, and nucleus. We successfully obtained the knockout mutants of above 15 *Hsps* by the CRISPR/Cas9 gene editing system. Under natural high-temperature conditions, the mutants of eight *Hsps* showed reduced yield mainly due to the seed setting rate or grain weight. Moreover, the rice quality of most of these mutants also changed, including increased grain chalkiness, decreased amylose content, and elevated total protein content, and the expressions of starch metabolism-related genes in the endosperm of these mutants were disturbed compared to the wild type under natural high-temperature conditions. In conclusion, our study provided new insights into the HSP gene family and found that it plays an important role in the formation of rice quality and yield.

## 1. Introduction

Heat shock proteins (Hsps) are synthesized in organisms in response to elevated temperatures, and their production can also be triggered by other stressors such as cold, salinity, drought, water stress, and oxidative stress [1]. Hsps are highly conserved across both prokaryotes and eukaryotes, with homology between these domains reaching up to 65% [1,2]. Hsps localized in different organelles exhibit diverse biological functions [3,4,5]. As molecular chaperones, Hsps play crucial roles in the transport, folding, assembly, and localization of newly synthesized peptides, as well as in refolding and degrading denatured proteins [6]. In addition to their chaperone functions, Hsps are involved in maintaining signal transduction and other biological processes essential for tissue and organ differentiation and development [7,8,9,10].

Hsps can be classified into various groups based on molecular weight, amino acid sequence, cellular localization, and function [6]. They are primarily grouped into small Hsp (sHsp), Hsp40, Hsp60, Hsp70, Hsp90, and Hsp100 families based on molecular weight [5,11]. The functions of these groups differ due to their distinct conserved domains. The sHsps, with molecular weights from 12 to 42 kDa, contain an α-crystallin domain of approximately 80–100 amino acids and act as ATP-independent chaperones, preventing protein aggregation under stress [12,13]. Hsp40 proteins, with molecular weights of 40–50 kDa, contain a “J-domain” and are homologous to the *E. coli* DnaJ protein, which is why they are also called the DnaJ subfamily [10]. Based on the presence or absence of a Zinc-finger domain and key HPD (His-Pro-Asp) tripeptides, the Hsp40 subfamily is further categorized into three subgroups: DnaJA, DnaJB, and DnaJC [10,14]. Hsp40 proteins serve as co-chaperones for the HSP70 family, enhancing their ATPase activity [15]. Hsp60, with a molecular weight of 60 kDa, includes the GroEL domain and exists in two forms: one found in bacteria, mitochondria, and chloroplasts (GroE/Cpn60), and the other in the cytoplasm of archaea and eukaryotes (TCP-1) [16]. Hsp60 proteins rely on energy for peptide synthesis and folding under stress [17,18,19]. The Hsp70 family (DnaK in bacteria) is one of the most thoroughly studied chaperone groups, featuring a conserved N-terminal ATPase domain (~44 kDa), a substrate-binding domain (~18 kDa), and a C-terminal “lid” (~10 kDa) [20]. Some members of the Hsp70 family, known as Hsp110, exhibit molecular weights of 100–170 kDa due to an acidic insertion or extended C-terminus [21,22]. Hsp90 proteins have three main structural domains: a highly conserved N-terminal ATPase domain, an intermediate domain, and a C-terminal domain featuring a dimeric interface [23]. These proteins play crucial roles in stress signal transduction, maintaining protein activity under stress conditions. Finally, Hsp100 proteins (also known as ClpB) have molecular weights of ~100 kDa, consisting of three distinct regions, the N-terminal domain, the ATPase domain, and the C-terminal domain, and are involved in protein folding and depolymerization [24,25,26]. Different Hsp proteins work together to form a network responsible for protein folding, translocation, degradation, and stress response, thus supporting normal growth and development under stress [27].

The biological functions of many Hsp genes have been identified across various species, with a significant impact on plant growth, especially under environmental stress. For instance, AtHSP17.4 is essential for seed maturation in *Arabidopsis* [28], while GhHSP24.7 in cotton regulates seed germination in a temperature-dependent manner [29]. The AtDjC21 and AtDjC29 proteins are vital for *Arabidopsis* growth, with mutations leading to embryo lethality [30]. *Arabidopsis* Hsp40 family member TMS1 is critical for pollen tube thermotolerance [31]. Enhanced expression of multiple Hsp70 proteins promotes bud burst in dormant poplar trees [32], and HSP70 (b-70) aids in gliadin assembly in maize endosperm [33]. Tobacco nbHSP90 regulates apoptosis signals to mitochondria [34]. In wheat, interference with HSP90.2 reduces leaf photosynthesis, grain weight, and yield, while its overexpression increases grain weight [35]. HSP90.3 in *Arabidopsis* mediates temperature-dependent defense responses [36], and HSP101 regulates chloroplast development [37]. These findings suggest that Hsps perform diverse biological roles in plant growth, development, and stress responses. In rice, four Hsp subfamilies have been identified, including 23 *sHsp* genes, 104 *Hsp40* genes, 32 *Hsp70* genes, and 3 *Hsp100* genes [9,10,20,26]. However, the function of only a few rice *Hsp* genes has been reported. For example, *DjA7* and *DjA8* are involved in chloroplast development [38], and *OSHSP40* regulates pollen development and seed setting [39]. Overexpression of *OsHsp17.0* and *OsHsp23.7* enhances drought and salt tolerance [40,41]. The Hsp60 family member *TCD9* influences chloroplast development under high temperatures [42], while *HSP60-3B* regulates male fertility by affecting starch particle formation [43]. Rice *cpHsp70-2* plays a key role in amyloplast development and chalkiness regulation under high-temperature stress [44,45], and *mtHsp70-1* inhibits heat-induced programmed cell death [46]. Finally, *Hsp101* is involved in starch biosynthesis and endosperm development under high temperatures [47]. However, the panoramic study of rice Hsp family genes is still lacking.

In order to fully understand the functions of rice Hsp family genes, especially their functions in regulating rice yield and quality, we carried out a comprehensive study on the molecular biochemical characteristics and biological functions of rice HSP family genes. We identified 22 more Hsp60 subfamily members and 8 more Hsp90 subfamily members, along with the reported members from 4 subfamilies; there are a total of 192 *Hsp* genes in rice across 6 subfamilies. The bioinformatics of these rice Hsps were performed. Subsequently, we conducted an in-depth analysis of 15 representative genes from five subfamilies. Using CRISPR/Cas9, we obtained the mutants for these 15 genes and evaluated yield and quality traits. We found that loss-of-function of most of these *Hsp* genes resulted in reduced yield and quality; they can regulate starch synthesis by influencing the expression of starch synthesis-related genes, highlighting their essential role in rice seed development and stress response. Our findings provide new insights into the mechanism of *Hsps* regulation of rice yield and quality, as well as a new theoretical basis and gene resources for the genetic improvement of rice yield and quality.

## 2. Results

### 2.1. Classification, Distribution, and Gene Structural Analysis of Hsp Family in Rice

**Figure 1:**
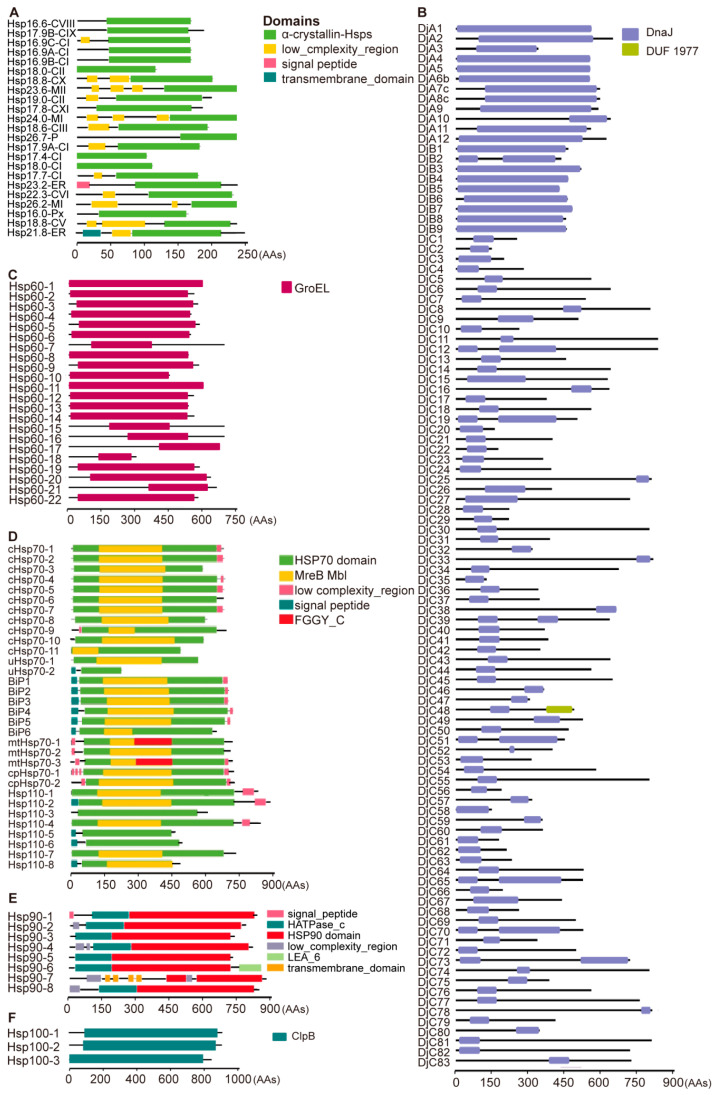
Conservative domains of 6 Hsp subfamilies in rice: (**A**) 23 small Hsp (sHsp) members: protein name suffix is subcellular localization, and each protein has the α-crystallin domain, which is the characteristic domain of sHsp; (**B**) 104 Hsp40 members, including three types, A (DjA1-DjA12), B (DjB1-DjB9), and C (DjC1-DjC83); each member has the DnaJ domain; (**C**) 22 Hsp60 members: each protein has a GroEL domain, and these proteins were named according to their positions on the chromosome; (**D**) 32 Hsp70 members, including 18 proteins whose name contain Hsp70, 6 Bips (Binding immunoglobulin proteins), and 8 Hsp110 members, each with an Hsp70 domain; (**E**) 8 Hsp90 members, each containing the Hsp90 domain; (**F**) 3 Hsp100 members, each harboring the ClpB domain specific to Hsp100. The scale at the bottom represents the number of amino acids. The conserved domain data of sHsp, Hsp70, and Hsp90 were downloaded from the Pfam database and visualized using the TBtools-II software. For Hsp40, Hsp60, and Hsp100, the conserved domain data were obtained from NCBI and visualized using a website.

We found that the rice genome contains a total of 192 Hsps, categorized into six subfamilies based on molecular weight and amino acid sequence: sHsp, Hsp40, Hsp60, Hsp70, Hsp90, and Hsp100, each with 23, 104, 22, 32, 8, and 3 members, respectively (Figure 1). Each subfamily has distinct conserved domains. The sHsp protein has an α-crystalline protein domain (Figure 1A). Hsp40 members contain a Dnaj domain structure and are further classified into three types: A (DjA1–DjA12), B (DjB1–DjB9), and C (DjC1–DjC83) (Figure 1B). Hsp60 proteins have a GroEL domain (Figure 1C). The Hsp70 subfamily consists of thirty-two members, including eighteen Hsp70 members, six Bip (Binding immunoglobulin Protein) members, and eight Hsp110 members, each of which contains domains of NBD, SBD, and the C-terminal “lid” (Figure 1D). Hsp90 subfamily members harbor N-terminal ATPase, an intermediate, and a C-terminal domain (Figure 1E). Hsp100 proteins contain the ClpB domain (Figure 1F). Conserved motifs were identified within subfamilies (Figure S1).

**Figure 2:**
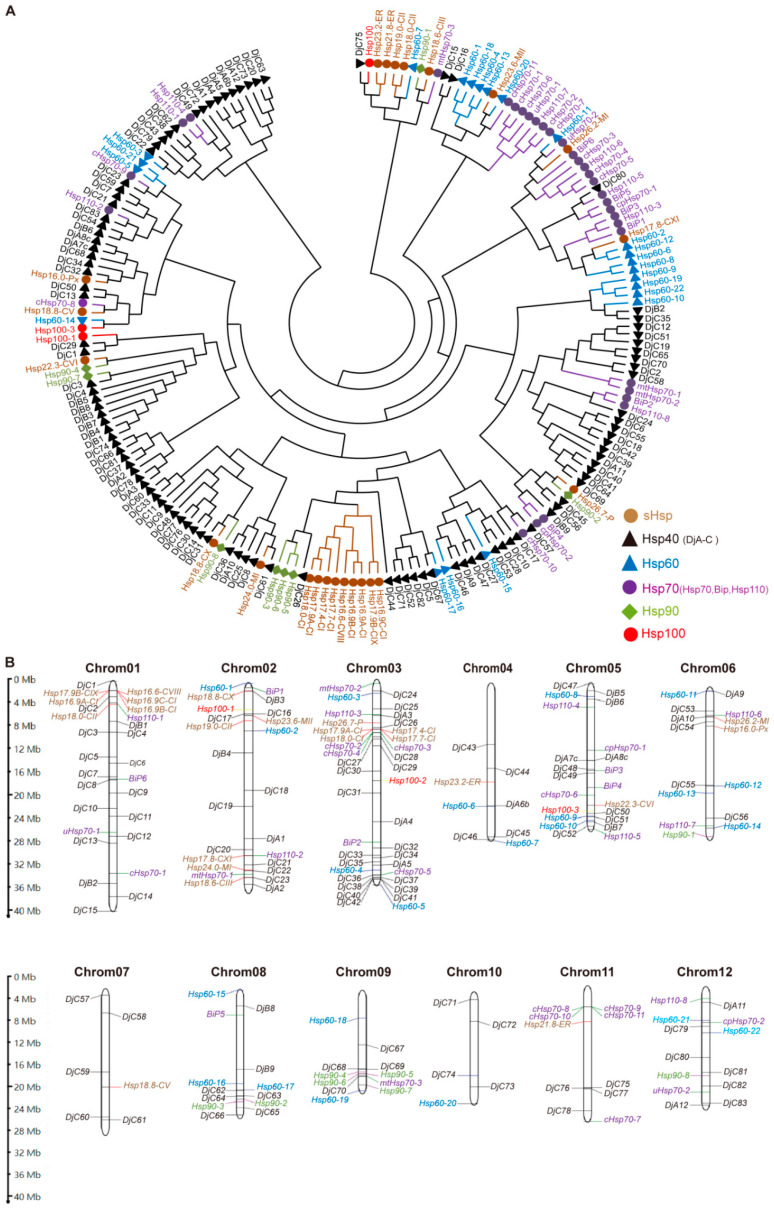
Phylogenetic tree and chromosomal localization analysis of 6 Hsp subfamilies. (**A**) Phylogenetic tree illustrating relationships among 192 Hsps. (**B**) Chromosomal distribution of 192 *Hsp* genes. The scale on the left is chromosome physical distance.

**Figure 3:**
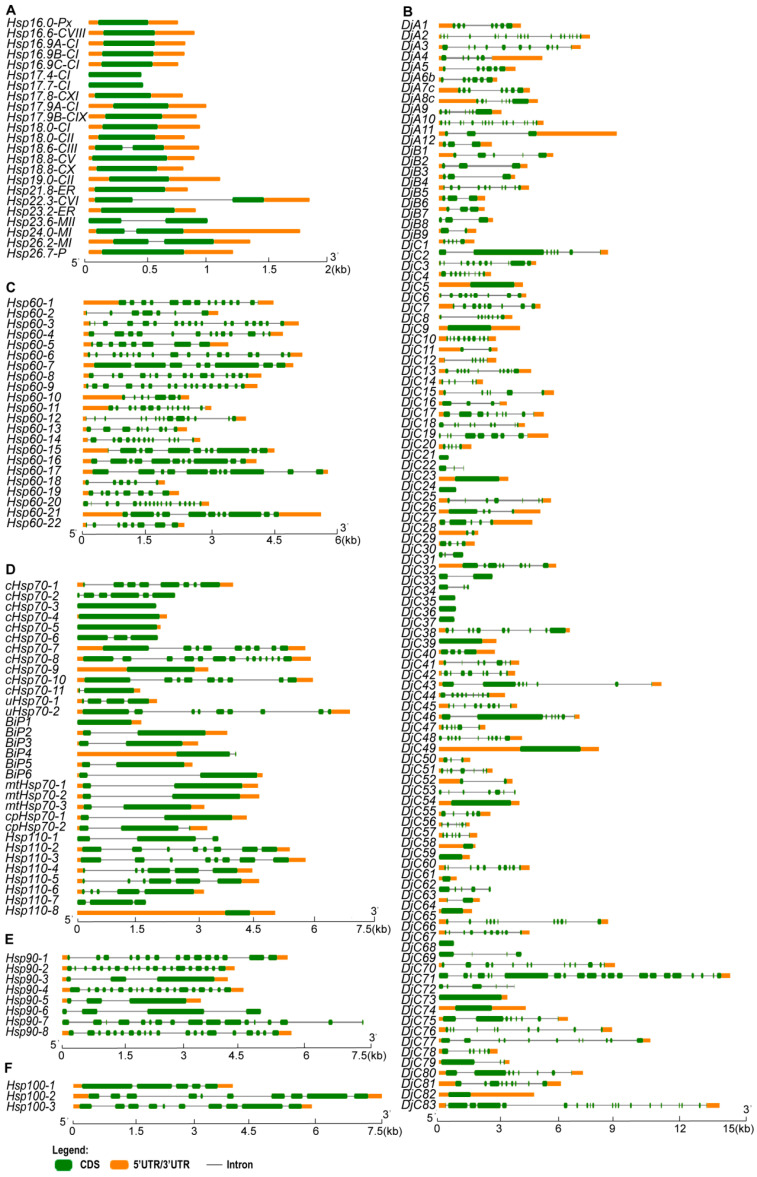
Gene structure of 192 *Hsp* genes in rice. (**A**–**F**) Gene structure depicting *sHsp*, *Hsp40*, *Hsp60*, *Hsp70*, *Hsp90*, and *Hsp100*. CDS indicates coding sequence for protein; UTR denotes untranslated region. The scale is the number of gene bases.

Phylogenetic analysis using aligned amino acid sequences showed that most subfamily members cluster together (Figure 2A), with sHsps concentrated in two regions and Hsp70 members in a single group. Hsp40, the largest subfamily, showed interspersion with other families. This analysis highlighted the close evolutionary relationships among different Hsp subfamilies. The 192 *Hsp* genes are distributed across all 12 chromosomes, with higher concentrations on chromosomes 1, 2, 3, and 5 (Figure 2B). Gene structure analysis revealed diversity in intron/exon numbers among subfamilies (Figure 3). Most *sHsp* genes (78%) lacked introns, while *Hsp60* and *Hsp100* exhibited multiple introns. Variations were seen in *Hsp40*, *Hsp70*, and *Hsp90* members, with some genes containing up to a dozen introns.

### 2.2. Differential Expression and Subcellular Localization of Hsp Genes

**Figure 4:**
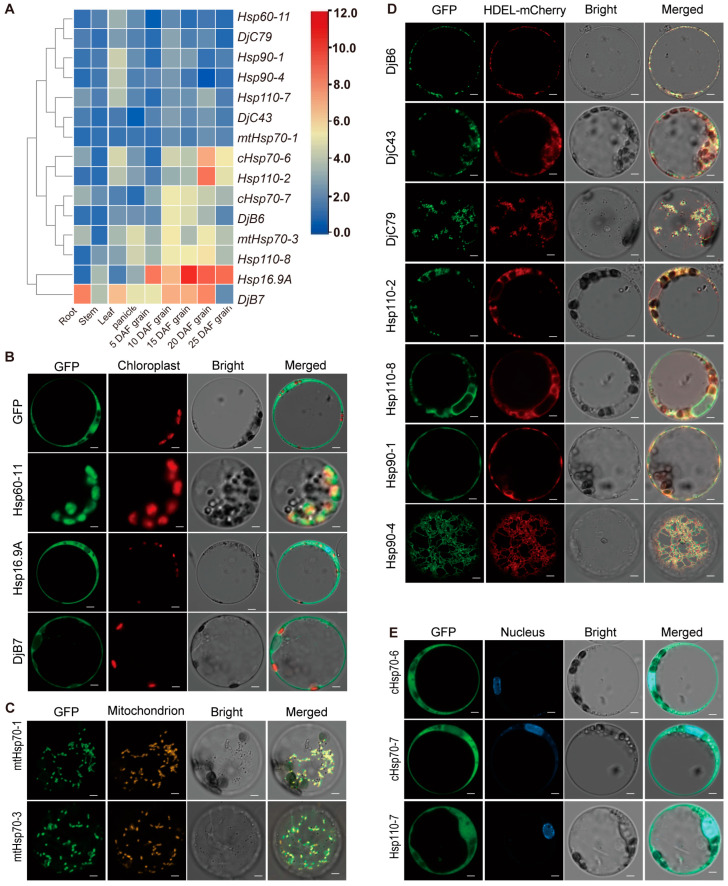
Expression pattern of 15 *Hsp* genes and subcellular localization analysis of 15 Hsp proteins. (**A**) The relative expression levels of 15 *Hsps* in roots, stems, leaves, panicles, and seeds at 5, 10, 15, 20, and 25 days after flowering. The heat map shows the expression level determined by TBtools-II (https://github.com/CJ-Chen/TBtools-II, (accessed on 31 March 2024)); the range of blue to red indicates the expression levels from low to high. Clustering is according to the expression level in each tissue. (**B**–**E**) Subcellular localization of Hsp proteins. Free green fluorescent protein (GFP) and full-length Hsp fusion proteins (Hsp-GFP) were transiently expressed in rice protoplasts. Hsp60-11-GFP co-localized with the chloroplast autofluorescence, Hsp16.9A and DjB7 localized in the cytoplasm (**B**). mtHsp70-1 and mtHsp70-3 co-localized with mitochondria, and the yellow signal represents the mitochondria dyed by Mito-Tracker Red (**C**). DjB6, DjC43, DjC79, Hsp110-2, Hsp110-8, Hsp90-1, and Hsp90-4 co-localized with HDEL-mCherry signals of the endoplasmic reticulum (**D**). cHsp70-6, cHsp70-7 and Hsp110-7 co-localized with the cytoplasm and DAPI signals of the nucleus (**E**). Scale bars, 5 μm.

Based on the predicted expression patterns from the website https://www.ricedata.cn/gene/index.htm (accessed on 19 April 2024), the 192 *Hsp* genes can be categorized into three groups (Figure S2): generally high expression in almost all tissues, high tissue-specific expression, and generally low expression in almost all tissues. We selected 15 representative genes from five subfamilies for further functional characterization analysis. RT-qPCR confirmed that the expression levels of all 15 genes were high in 10d grains after flowering; 8 genes (*Hsp16.9A*, *DjB6*, *DjB7*, *mtHsp70-3*, *cHsp70-6*, *cHsp70-7*, *Hsp110-2*, and *Hsp110-8*) exhibited high expression levels in grains of multiple periods (Figure 4A). It is evident that these *Hsp* genes exhibit a trend of preferential expression in grains.

The subcellular localization of Hsp proteins was examined using HSP-GFP fusion constructs in rice protoplasts (Figure 4B–E). Among the 15 Hsp proteins, 7 proteins (DjB6, DjC43, DjC79, Hsp110-2, Hsp110-8, Hsp90-1, and Hsp90-4) from three subfamilies were co-localized with the HDEL-mCherry signal of the endoplasmic reticulum (ER) (Figure 4D). Hsp60-11 co-localized with chloroplasts (Figure 4B). Hsp16.9A and DjB7 localized in the cytoplasm (Figure 4B). mtHsp70-1 and mtHsp70-3 co-localized with mitochondria (Figure 4C). Additionally, three proteins (cHsp70-6, cHsp70-7, and Hsp110-7) co-localized with DAPI signals of the nucleus and cytoplasm (Figure 4E).

### 2.3. The Expression Levels of Hsp Genes Are More Easily and Strongly Induced by Heat Stress

**Figure 5:**
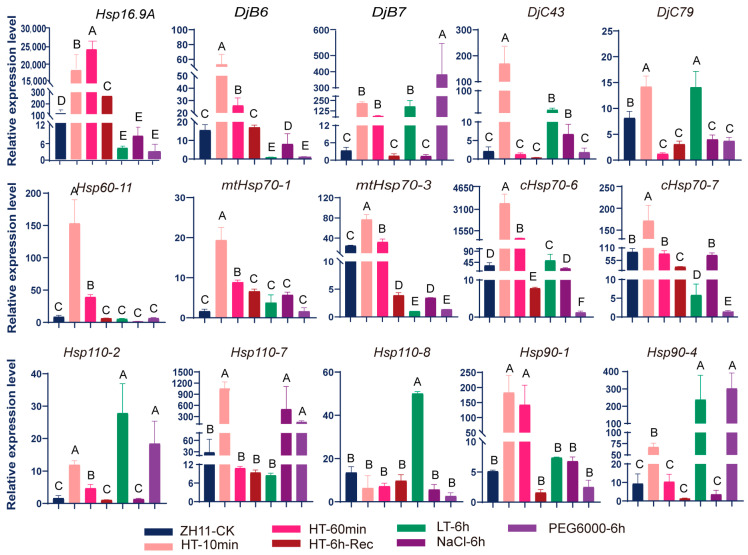
The induced expression levels of 15 *Hsp* genes in the seedlings of ZH11 under different stresses. ZH11-CK represents the normal seedlings as control with no treatment. HT-10min and HT-60min represent the seedlings that were moved to a high temperature of 42 °C from normal temperature for 10 and 60 min. HT-6h-Rec represents the seedlings that recovered at a normal temperature for 6 h after 60 min of heat shock. LT-6h represents the seedlings that were treated at 5 °C for 6 h. NaCl-6h represents the seedlings that were treated with high salt stress for 6 h (100 mmol/L NaCl). PEG6000-6h represents the seedlings that were treated at 20% PEG stress for 6 h. The expression levels of *Hsps* were detected in the above treated seedlings and ZH11-CK. Data are means ± SD (n = 3). Different letters indicate significant differences at *p* < 0.05 by ANOVA and Duncan’s test.

To investigate the response of *Hsp* genes to adversity stresses, the induced expression levels of 15 genes under high-temperature, low-temperature, high-salt, and drought conditions were assessed by the RT-qPCR technique. The results showed that 14 genes exhibited increased expression under high temperatures, the expression levels of 7 genes were upregulated by low temperatures, 2 gene (*DjC43* and *Hsp110-7*) were upregulated in high-salt conditions, and 4 genes (*DjB7*, *Hsp110-2*, *Hsp110-7*, and *Hsp90-4*) showed increased expression under drought stress (Figure 5). This indicates that some *Hsp* genes were also induced by other stresses except for heat stress, though the induced expression of the same gene under high-temperature conditions was significantly higher than that under other stresses. Specifically, high temperature resulted in a hundreds-of-fold increase in the synthesis of *Hsp16.9A* and *cHsp70-6* within just 10 min. The expression of *DjB7*, *DjC43*, *Hsp60-11*, *mtHsp70-1*, *Hsp110-7*, and *hsp90-1* increased dozens of times under heat stress. While the expression of only one gene (*Hsp90-4*) exhibited a 100-fold increase under other stress conditions (drought), the expression of other genes increased by twenty or several times at most.

After high-temperature treatment of rice seedlings, the transcriptome analysis showed massive upregulation of thousands of genes, with *Hsp* genes most significantly enriched in the “protein processing in the endoplasmic reticulum” pathway (Figure S3A–F). At the same time, the expression of other *Hsp* genes was also upregulated in multiple KEGG enrichment pathways (Figure S3G), including ‘plant and pathogen interaction’, ‘RNA degradation’, ‘Spliceosome’, and ‘Endocytosis’. It can be inferred that under heat stress, Hsp proteins may play an important role.

### 2.4. Creation of CRISPR Mutants

To identify the biological function of the 15 *Hsp* genes (1 *sHsp*, 4 *Hsp40*, 1 *Hsp60*, 7 *Hsp70*, and 2 *Hsp90* genes), their mutants were generated by the CRISPR/Cas9 gene editing system in a *japonica* rice cultivar ZH11. To completely turn off the gene function in the mutants, target sites for gene editing were located in the front exon region of each gene (Figure S4A). It was observed that most mutants of 14 genes exhibited early termination of the translation process; 1 gene exhibited the amino acid replacement in its two lines (Figure S4B). Just one line was obtained for genes *DjB6* and *Hsp90-4*; one or two lines of each gene were studied further. Among the 15 genes, the analogs of gene editing target sites of *Hsp16.9A*, *cHsp70-6*, and *cHsp70-7* also appear on other homologous genes, but the corresponding sites of these homologous genes were not mutated (Figure S5); the gene editing target sites of the other 12 genes have good specificity, and there are no analogs in their homologous genes. So, stable homozygous gene editing mutants were obtained for the 15 genes.

### 2.5. Hsp Genes Regulate Rice Yield

**Figure 6:**
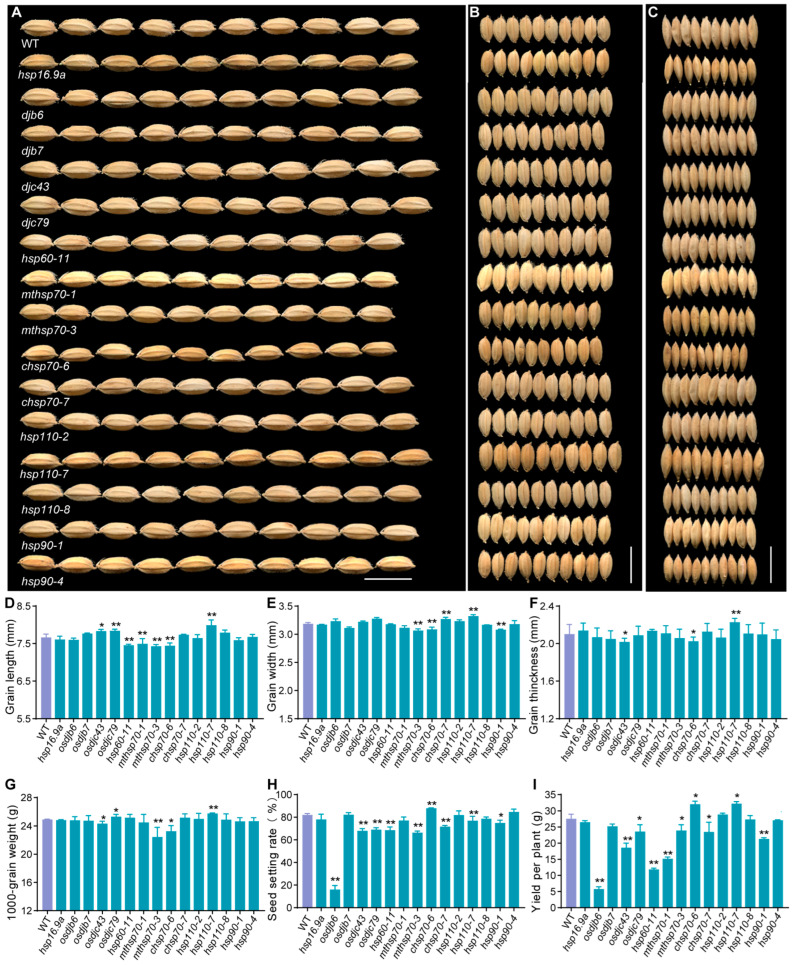
Grain size- and yield-related traits of ZH11 and the *hsp* mutants. (**A**–**C**) Comparison of the grain length (**A**), grain width (**B**), and grain thickness (**C**) of ZH11 and *hsp* mutants; scale bars = 1 cm. (**D**–**I**) Grain length (**D**), grain width (**E**), grain thickness (**F**), 1000-grain weight (**G**), seed setting rate (**H**), and yield per plant (**I**) of ZH11 and the mutants. The investigated plants were grown in natural high-temperature conditions in fields in Hangzhou in 2023. Data are means ± SD; n = 20 in (**D**–**F**), n = 3 in (**G**), and n = 10 in (**H**,**I**), and no less than 200 grains per replication in (**G**). Asterisks show statistical significance between the WT and the mutants, as determined by Student’s *t*-test (* *p* < 0.05; ** *p* < 0.01).

To investigate whether *Hsp* genes regulate rice yield, the mutants of 15 *Hsp* genes and the wild type were grown under natural high-temperature conditions. No significant differences in plant morphology, such as plant height and tiller number, were observed between mutants and wild-type plants (Figure S6A–C). The growth periods of the wild type and the mutants were also consistent (Figure S6D). Compared to the wild type, the grain shape, grain weight, and seed setting rate showed changes in the mutants. Among the 15 mutants, there were 7 mutants exhibiting changes in grain length, including *djc43*, *djc79*, *hsp60-11*, *mthsp70-1*, *mthsp70-3*, *chsp70-6*, and *hsp110-7* (Figure 6A)*;* 5 mutants exhibited changes in grain width, such as *mthsp70-3*, *chsp70-6*, *chsp70-7*, *hsp110-7*, and *hsp90-1* (Figure 6B); there were 3 mutants exhibiting changes on grain thickness, for instance, *djc43*, *mthsp70-3*, and *hsp110-7* (Figure 6C); and 5 mutants exhibited changes on 1000-grain weight, including *djc43*, *djc79*, *mthsp70-3*, *chsp70-6*, and *hsp110-7* (Figure 6D). Except for the changes in grain size, the seed setting rate of nine mutants changed, with eight significantly decreased and one increased (Figure 6H). And finally, the smaller size and reduced seed setting rate of mutants *djc43*, *hsp60-11*, *mthsp70-3*, and *hsp90-1* led to a decreased yield per plant. For mutants *djc79* and *chsp70-7*, the grain size became larger, but the seed setting rate decreased, resulting in a decreased yield per plant. The yield per plant of *mthsp70-1* also reduced due to the smaller grain length, with no changes in seed setting rate. Only two mutants showed an increase in yield per plant (*hsp110-7* and *chsp70-6*); for mutant *hsp110-7*, the seed setting rate reduced from 82.13% to 77.00%, but grain length, width, thickness, and 1000-grain weight all increased significantly, resulting in an increased yield per plant. For mutant *chsp70-6*, the grain length, width, thickness, and 1000-grain weight all decreased, but the seed setting rate increased from 82.13% to 88.05%, resulting in an increased yield per plant. These results indicate that the loss-of-function of most *Hsp* genes leads to changes in grain size, reduced seed setting rate, and decreased yield per plant under natural high-temperature conditions.

### 2.6. Hsp Genes Regulate Rice Quality

**Figure 7:**
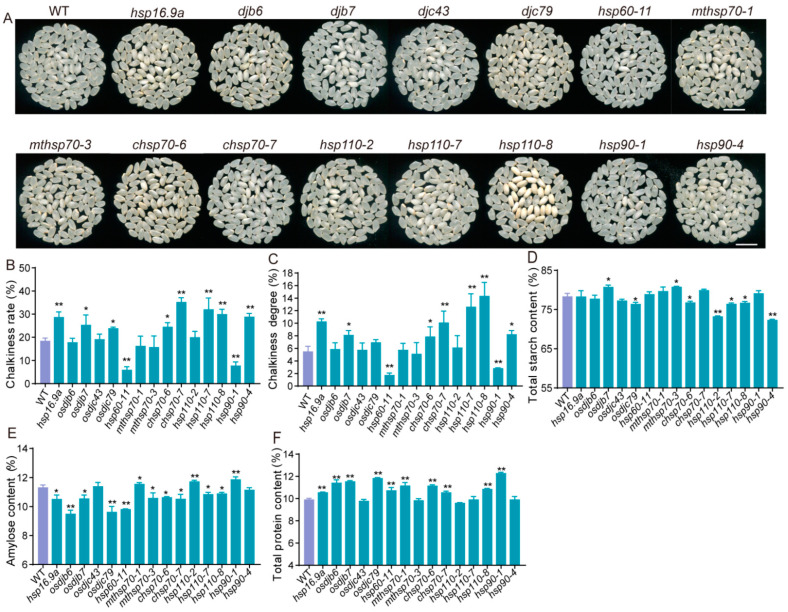
Grain quality of ZH11 and *hsp* mutants under natural high temperature in the Hangzhou field in 2023. (**A**) Appearance of mature grains of ZH11 and 15 *hsp* mutants. Scale bars = 1 cm. (**B**,**C**) Chalkiness rate and chalkiness degree of ZH11 and 15 *hsp* mutants grains. (**D**–**F**) Total starch, amylose, and total protein contents of ZH11 and the mutant grains. Data are means ± SD (n = 3); no less than 200 grains per replication in (**B**,**C**). Asterisks show the statistical significance between the WT and the mutants, as determined by Student’s *t*-test (* *p* < 0.05; ** *p* < 0.01).

We also compared the rice quality of the mutants of 15 *Hsps*. When grown under natural high-temperature conditions, mutants of eight *Hsps* (*hsp16.9a*, *djb7*, *djc79*, *chsp70-6*, *chsp70-7*, *hsp110-7*, *hsp110-8*, and *hsp90-4*) exhibited an increased chalkiness rate and degree, while two mutants (*hsp60-11* and *hsp90-1*) displayed a decreased chalkiness rate and degree (Figure 7A–C). Subsequently, the contents of storage substances in the mature endosperm were measured. Compared to the wild type, the total starch content decreased in mutants *djc79*, *chsp70-6*, *hsp110-2*, *hsp110-7*, *hsp110-8*, and *hsp90-4*, while it increased in mutants *djb7* and *mthsp70-3* (Figure 7D). Additionally, the amylose content decreased in 10 mutants (*hsp16.9a*, *djb6*, *djb7*, *djc79*, *hsp60-11*, *mthsp70-3*, *chsp70-6*, *chsp70-7*, *hsp110-7* and *hsp110-8*), whereas it increased in 3 mutants (*mthsp70-1*, *hsp110-2* and *hsp90-1*) (Figure 7E). Furthermore, the total protein content increased in 10 mutants (*hsp16.9a*, *djb6*, *djb7*, *djc79*, *hsp60-11*, *mthsp70-1*, *chsp70-6*, *chsp70-7*, *hsp110-8*, and *hsp90-1*) (Figure 7F). These results indicated that the mutation of these *Hsp* genes leads to an increased rate of chalkiness. In these mutants, at least one component of seed amylose or total starch content was reduced, while total protein content was elevated.

Furthermore, the plants of ZH11 and all mutants at the filling stage were subjected to high temperatures in the greenhouse. The high temperature was set at 28/34 °C for a duration of 15 days; the normal temperature was set at 22 °C/28 °C, and then the chalkiness phenotype was investigated. The results showed that under high temperatures, both ZH11 and all mutants showed increased chalkiness rate and chalkiness degree. Among these mutants, 13 mutants showed higher changes in chalkiness rate or/and chalkiness degree than ZH11 after high-temperature stress (Figure S8, Table S2). Overall, these *Hsp* genes play an important role in regulating rice quality under high-temperature conditions.

### 2.7. Hsp Genes Affect Starch Synthesis Pathway Under Naturally High Temperature

**Figure 8:**
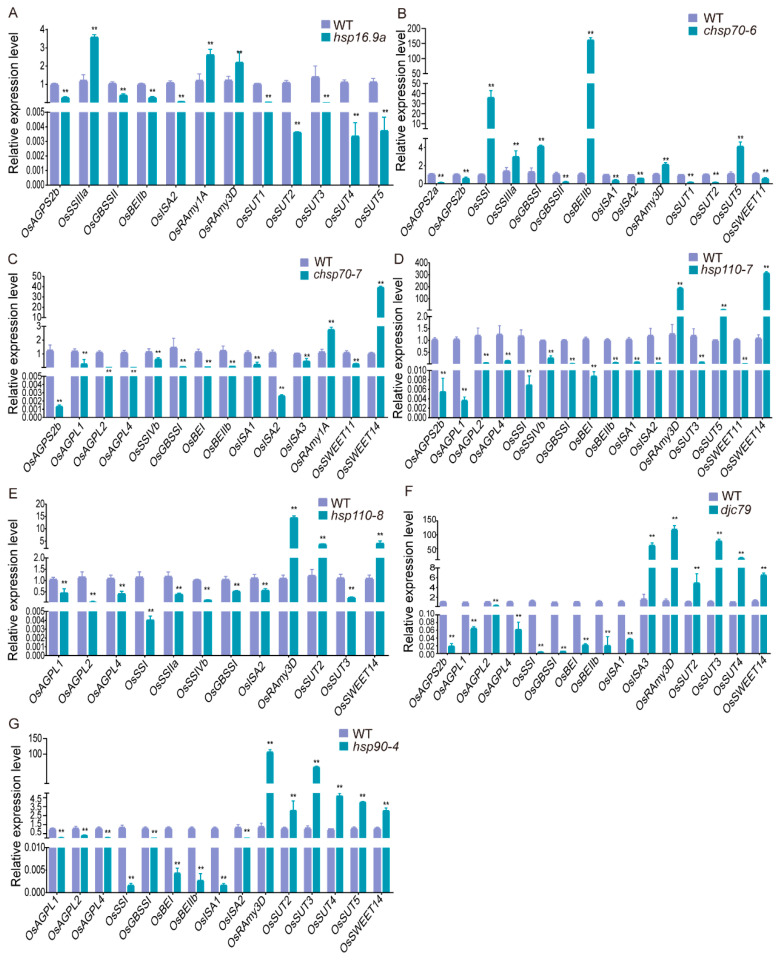
The expression levels of starch synthesis-related genes in developing endosperm of the wild type (WT) and 7 mutants (*hsp16.9a*, *chsp70-6*, *chsp70-7*, *hsp110-7*, *hsp110-8*, *djc79*, and *hsp90-4*). (**A**–**G**) The relative expression level of starch synthesis-related genes in endosperm at 10d after flowering under natural high temperature in the Hangzhou field in 2023. The data presented here are the relative expression levels of the genes that are expressed differently between mutants and the wild type. The rice UBIQUITIN gene was used as the internal control. Data are means ± SD of three individual replicates. Asterisks show the statistical significance between the WT and the mutants, as determined by Student’s *t*-test (** *p* < 0.01).

The starch in rice endosperm rapidly accumulates 5–10 days after flowering; the chalkiness phenotype of grains is closely related to the obstruction of starch synthesis [47]. Based on the observed chalkiness phenotype of the *hsp* mutants, we selected 29 major starch metabolism-related genes, including 13 starch synthesis genes (*OsAGPS1*, *OsAGPS2a*, *OsAGPS2b*, *OsAGPL1*, *OsAGPL2*, *OsAGPL4*, *OsSSI*, *OsSSIIa*, *OsSSIIIa*, *OsSSIIIb*, *OsSSIVb*, *OsGBSSI*, and *OsGBSSII*), eight starch degradation genes (*OsBEI*, *OsBEIIa*, *OsBEIIb*, *OsISA1*-*OsISA3*, *OsRAmy1A*, and *OsRAmy3D*), and eight sugar transport genes (*OsSUT1*-*OsSUT5*, *OsSWEET11*, *OsSWEET13*, and *OsSWEET14*); the expression of these genes was analyzed in 10d endosperm of ZH11 and seven mutants (*hsp16.9a*, *chsp70-6*, *chsp70-7*, *hsp110-7*, *hsp110-8*, *djc79*, and *hsp90-4*) exhibiting an increased chalkiness rate under high temperature (28/34 °C). These mutants were categorized into two groups according to the changes in the nutrient content in seeds (Figure 8).

The first category consists of mutants *hsp16.9a*, *chsp70-6*, *chsp70-7*, and *hsp110-7* (Figure 8A–D), with reduced amylose contents. The expression of *OsAGPS2b* decreased, the expression of *OsGBSSI* and *OsGBSSII* decreased, and the expression of *OsRAmy1A* and *OsRAmy3D* increased. The second category, including three mutants (Figure 8E–G) *hsp110-8*, *djc79*, and *hsp90-4*, exhibited decreased total starch content in their seeds. Compared with those in ZH11, the expression levels of *OsAGPL1*, *OsAGPL2*, and *OsAGPL4* all significantly decreased, and the expression of *OsRAmy1A* and *OsRAmy3D*, along with *OsSUT2* and *OsSWEET14*, increased in the three mutants. Furthermore, mutants *djc79* and *hsp110-8*, with reduced amylose contents, exhibited decreased expression of the *OsSSI* and *OsGBSSI* genes. Taken together, these mutants showed altered expression of key starch synthesis and degradation genes, confirming the impact of *hsp* mutations on starch accumulation and chalkiness formation.

## 3. Discussion

### 3.1. The Diversity and Abundance of Hsp Genes Are Notable Across Species

*Hsp* gene families have been extensively identified across various species, including both animals and plants. In animals, the bovine genome contains a total of 67 *Hsp* genes, comprising 10 *sHsp* genes, 43 *Hsp40* genes, 10 *Hsp70* genes, and 4 *Hsp90* genes [48]. A total of 47 *Hsp* genes have been identified in A. *glabripennis*, including 21 *sHsp* genes, 9 *Hsp60* genes, 14 *Hsp70* genes, and 3 *Hsp90* genes [49]. The whitefly has 26 *Hsp* genes, including 3 *Hsp90* genes, 17 *Hsp70* genes, 1 *Hsp60* gene, and 5 *sHsp* genes [50]. The apple snail possesses 42 *Hsp* genes, including 12 *sHsp* genes, 13 *Hsp40* and *Hsp70* genes, 1 *Hsp60* gene, and 3 *Hsp90* genes [51]. Additionally, the Hsp family genes has been analyzed in various plant species. In *Arabidopsis*, six *Hsp90* genes and three *Hsp100* genes have been identified [37,52]. In maize, 99 *DnaJ* and 22 *Hsp70* genes have been identified through whole-genome analysis [53,54]. Other crop species exhibit considerable variability in *Hsp* gene abundance: wheat has 7 *sHsp* genes, barley has 13 *sHsp* genes [55], and sorghum has 113 *Hsp40/DnaJ* genes [56]. In potatoes, 48 *sHsp* genes have been identified [57], while in soybeans, 51 *sHsp* genes are considered likely candidates [58]. In horticultural crops, the grape genome contains 48 *sHsp* genes and 78 *DnaJ* genes [59,60]. The *Ziziphus jujuba* has 21 *Hsp70* genes and 6 *Hsp100* genes [61]. In rice, four Hsp subfamilies have been identified, including 23 *sHsp* genes, 104 *Hsp40* genes, 32 *Hsp70* genes, and 3 *Hsp100* genes [9,10,20,26]. In this study, we identified 22 more rice *Hsp60* genes and 8 *Hsp90* members (Figure 1C,E). So far, the number of identified rice *Hsp* genes is up to 192. These findings demonstrate that *Hsp* genes are highly abundant and diverse across both animals and plants; among them, *Hsp* is the most abundant in rice. Given this diversity, *Hsps* play multifaceted roles across different organisms, warranting further investigation into their functional diversity.

### 3.2. The Functions of Hsps Are Crucial for Seed Biological Growth and Development

The Hsp family genes play a fundamental role in regulating plant growth and development, and the loss of these genes impacts both yield-related and quality-related traits [62,63,64,65,66,67]. Previous studies have shown that rice *Hsp* genes *HSP60-3B* can regulate seed setting by influencing starch biosynthesis in male gametophytes [43]. Rice *cHsp70-2* and *Hsp101* can positively regulate rice grain width and 1000-grain weight [44,47], but the underlying mechanisms remain unclear. HSP90.2 regulates wheat yield by affecting photosynthetic capacity [35,68]. Other studies showed that genes may influence cell number or size by influencing cytokinin metabolism, thereby regulating grain size [69,70,71]. In this study, five genes (*Hsp60-11*, *mtHsp70-1*, *mtHsp70-3*, *cHsp70-6*, and *Hsp90-1*) positively regulate rice grain size, while *DjC79* and *Hsp110-7* have a negative regulatory function (Figure 6D–F); eight genes (*DjB6*, *DjC43*, *DjC79*, *Hsp60-11*, *mtHsp70-1*, *mtHsp70-3*, *cHsp70-7*, and *Hsp90-1*) positively regulate rice seed setting rate and yield per plant, while *cHsp70-6* plays a negative regulatory role (Figure 6H–I). These results indicate that rice *Hsp* genes regulate grain size and seed setting rate, ultimately affecting yield.

Notably, the mutants of 15 *Hsp* genes not only changed the yield-related traits but also the quality-related indicators. Rice *OsbZIP60/O3* can regulate the chalkiness phenotype by affecting the expression of starch synthesis-related genes [72,73]. In this study, we found that *Hsp* genes regulate rice quality in the same way. The chalkiness rate of eight mutants increased under naturally high temperatures (Figure 7), and the content of total starch and/or amylose in the mutant seeds decreased, while the content of total protein remained unchanged or increased (Figure 7D–F). Then, the expression of starch synthesis-related genes was assessed in seven mutants exhibiting an increased chalkiness rate under naturally high temperatures. The results showed that the expression of starch synthesis genes decreased in the seven mutants tested, while genes responsible for starch degradation and sugar transport increased (Figure 8 and Figure S9), thus resulting in abnormal starch accumulation, leading to chalky grains. The results indicate that *Hsp* genes are critical for balancing starch synthesis and metabolism. This underscores the pivotal role of *Hsp* genes in seed filling and highlights their importance in maintaining both yield and quality, providing valuable genetic resources for future breeding programs aimed at producing high-yield, high-quality rice.

### 3.3. Hsp Genes Located in ER May Participate in Endosperm Development Under High Temperature

Global climate change gradually raises temperatures, leading to more frequent extreme heat events [2,74]. When temperatures exceed 37 °C for more than three consecutive days during the reproductive growth stage, it adversely affects pollen development and fertilization, reducing both yield and quality [75]. High temperature during the grain-filling stage in rice can induce endoplasmic reticulum stress, resulting in a chalkiness phenotype of seeds [76,77]. In our study, transcriptomic analysis revealed that the Hsp proteins responsible for protein processing in the endoplasmic reticulum can quickly respond to high temperatures by upregulating the expression of corresponding genes (Figure S3D–G). Furthermore, an in-depth study of the 15 *Hsp* genes found that the loss-of-function of 8 genes exhibited a more pronounced chalkiness phenotype after high-temperature treatment (Figure 7 and Figure S8, Table S2), suggesting that these *Hsp* genes are functionally involved in heat stress response, with seven proteins localized in the ER (Figure 4D). When plants are subjected to heat stress, many denatured proteins accumulate; the Hsp chaperone proteins are responsible for the folding, translocation, and degradation of denatured proteins [26,27]. The activation of the unfolded protein response (UPR) can be triggered by the upregulation of ER-localized Hsp70 members *Bip1*-*Bip5* [72,73]. It is speculated that in our study, the mutants with loss-of-function of the ER-localized Hsp proteins disrupt the activation of the UPR pathway, resulting in the accumulation of denatured proteins, and leading to the formation of chalky grains. The activation of the UPR can affect the storage of substances and the expression of starch synthesis-related genes [72,73]. In this study, the loss-of-function of the ER-localized proteins resulted in alterations in the content of stored substances (Figure 4D and Figure 7D–F). And the expression of starch synthesis-related genes was affected in all the tested mutants (Figure 8). It is reasonably hypothesized that Hsp proteins located in the ER likely participate in the UPR pathway to maintain ER homeostasis, ensuring the normal development of rice endosperm under high temperatures.

The pathways through which Hsps respond to stress in nature are highly complex. Future research could delve into these interactions between Hsps to better understand their collective role. The functions of Hsps under other stresses can also be studied, leveraging the cross-protection effect of Hsps, which may enhance plant resistance to multiple stresses, thereby improving yield and quality.

## 4. Materials and Methods

### 4.1. Identification and Phylogenetic Analysis of Hsp Genes on Rice

In previous studies, four Hsp subfamilies have been reported, including 23 *sHsp* genes, 104 *Hsp40* genes, 32 *Hsp70* genes, and 3 *Hsp100* genes [9,10,20,26]. To identify all members of the Hsp60 and Hsp90 subfamilies, homology searches were conducted based on previously reported *Hsp* genes. Genomic and protein sequence data were downloaded from the National Center for Biotechnology Information (NCBI, https://www.ncbi.nlm.nih.gov (accessed on 11 April 2024)). The conserved domain data of sHsp, Hsp70, and Hsp90 were sourced from the Pfam database and visualized using the TBtools-II software. For Hsp40, Hsp60, and Hsp100, the conserved domain data were obtained from the NCBI Conserved Domain Database and visualized using the tool (https://www.chiplot.online/conserved_domains_plot.html (accessed on 12 April 2024)). A phylogenetic tree was constructed using the Neighbor-Joining method in MEGA 11 software, enabling insights into the evolutionary relationships among these gene families.

### 4.2. Chromosomal Location and Gene Structure Analysis

The chromosomal locations of the identified Hsp genes were determined using the MG2C website (http://mg2c.iask.in/mg2c_v2.0 (accessed on 17 April 2024)). The genomic and coding sequence (CDS) data were downloaded from the website of NCBI, and the gene structure was visualized through the GSDS website (http://gsds.gao-lab.org/index.php (accessed on 17 April 2024)), illustrating the exon–intron configurations and gene models.

### 4.3. Gene Expression Profile Analysis

#### 4.3.1. Prediction of Expression Levels of 192 Genes Across Different Tissues

The expression patterns of 192 Hsp genes across various rice tissues were retrieved from the RiceData website (https://www.ricedata.cn/gene/index.htm (accessed on 25 April 2024)). These expression patterns were visualized using TBtools-II (accessed on 25 April 2024) software, providing a comprehensive overview of the tissue-specific expression of Hsp genes.

#### 4.3.2. Expression Analysis Across Different Tissues

Total RNAs were extracted from roots, stems, leaves, panicles, and developing grains 5, 10, 15, 20, and 25 days after flowering using the RNAprep Pure Plant Kit (DP441, TIANGEN, Beijing, China). The extracted RNA was reverse-transcribed into complementary DNA (cDNA) using the ReverTra Ace qPCR RT kit (Toyobo, Osaka, Japan). The gene expression patterns were evaluated through quantitative real-time PCR (RT-qPCR) using SYBR Green Real-Time PCR Master Mix (Toyobo, Osaka, Japan), with each sample repeated three times to ensure accuracy. The rice *Ubiquitin* gene (Os03g0234200) was used as an internal control. The RT-qPCR reaction system is 10uL in total, including 1 uL of upstream primer, 1 uL of downstream primer, 2 uL of cDNA, 5 uL of mix, and 1 uL of ddH_2_O. The qRT-PCR program and system were as follows: Step 1: 95 °C for 3 min; Step 2: 95 °C for 10 s, 58 °C for 15 s, 72 °C for 15 s, Step 2 for 45 cycles; Step 3: 72 °C for 2min. These expression patterns were visualized using TBtools-II software.

#### 4.3.3. Induced Expressions Under Different Stress Conditions

Two-week-old ZH11 seedlings were subjected to various stress conditions, including heat stress (42 °C for 10 and 60 min, followed by heat recovery for 6 h), cold stress (5 °C for 6 h), high salt stress (100 mmol/L NaCl for 6 h), and drought stress (20% PEG for 6 h). Gene expression levels under these stress conditions were examined using RT-qPCR, offering insights into the stress-responsive behavior of the Hsp genes.

#### 4.3.4. RNA Extraction and Transcriptome Analysis

For transcriptome analysis, two-week-old ZH11 seedlings were subjected to heat stress (42 °C for 10, 30, and 60 min). Total RNA was extracted and subjected to standard transcriptome sequencing procedures, including RNA purification, cDNA synthesis, adapter ligation, and PCR amplification. The raw sequence data were cleaned and mapped to the rice reference genome for further analysis.

### 4.4. Subcellular Localization Analysis

To examine the subcellular localization of the Hsps, their coding sequences without termination codons were cloned into the pYBA1132-GFP vector. Then, the fusion of GFP and Hsp proteins together with marker vectors (HDEL-mCherry) was co-transformed into the protoplast of rice. DAPI was used as the nuclear marker. The GFP control protein was evenly distributed in the cytoplasm and nucleus. Confocal laser scanning microscopy (Karl Zeiss, Jena, Germany) was employed to observe the fluorescence signals, enabling the determination of the precise subcellular localization of the Hsp proteins.

### 4.5. Plant Materials and Growth Conditions

The *japonica* rice cultivar Zhonghua 11 (ZH11) was used as the primary plant material in this study. ZH11 was cultivated by the Institute of Crops of the Chinese Academy of Agricultural Sciences in 1979 with Jingfeng No.5/Tetep/Fujin, and cultivated in 1984. Mutant plants were generated by the CRISPR/Cas9 gene editing system. The mutants and wild type (ZH11) were grown in the fields of Hangzhou in 2023; the average temperature outdoors in the daytime was 34 °C from 20 July 2023 to 20 August 2023, following standard agronomic practices. After flowering, some well-grown ZH11 and mutant plants were moved to plant growth chambers under normal-temperature (28 °C, 12 h light/22 °C, 12 h dark) and high-temperature (35 °C, 12 h light/28 °C, 12 h dark) conditions for 15 days, and then moved to normal-temperature conditions until the grains are ripe.

### 4.6. Methods of Phenotype Evaluation

Plant height is measured as the height of the highest part of the spike above the ground. The grain length, grain width, and grain thickness were measured by the Wanshen SC-G automatic seed testing and analysis system (Wseen, Hangzhou, China). Mature and plump grains were used to measure 1000-grain weight. The chalkiness rate and chalkiness degree were measured by the Wanshen SC-E Rice Quality Analyzer (Wseen, Hangzhou, China), with each sample repeated three times to ensure accuracy.

### 4.7. Determination of Total Starch, Amylose Starch, and Total Protein Contents in the Endosperm

The amylose starch and total starch contents of mature endosperm were measured using the Megazyme K-TSTA and K-AMYL starch assay kits. The total protein content was measured following previously established protocols [78].

### 4.8. Statistical Analysis

All statistical analyses were conducted using GraphPad Prism 9. Significant differences between the treatment and control groups were determined using t-tests and one-way ANOVA, followed by Duncan’s multiple comparisons, with *p*-values less than 0.05 or 0.01 considered statistically significant.

## 5. Conclusions

In this study, the Hsp family in rice was systematically analyzed and classified into six subfamilies: sHsp, Hsp40, Hsp60, Hsp70, Hsp90, and Hsp100. Bioinformatic analyses revealed a high degree of conservation within each subfamily, suggesting functional significance. Structural analysis, gene expression profiling, and subcellular localization studies indicated that these characteristics are likely correlated with their specialized roles in cellular processes. Notably, Hsps were shown to play a pivotal role in the grain-filling process, and the loss-of-function of most Hsps led to a decline in rice yield and quality, particularly under high-temperature conditions. Therefore, by introducing powerful *Hsp* genes into rice varieties through genetic engineering or traditional breeding methods, it may be possible to improve the yield and quality of rice, which is significant for meeting the increasing demand for food. In conclusion, our findings provide new insights into the mechanism of heat protein regulation of rice yield and quality, and provide a new theoretical basis and gene resources for the genetic improvement of rice yield and quality.

## Data Availability

The datasets supporting the conclusions of this article are included within the article and Supplementary Materials.

## Supplementary Materials

The following supporting information can be downloaded at https://www.mdpi.com/article/10.3390/ijms252211931/s1.
