# Supplementary material for: Genome-Wide Analysis of Heat Shock Protein Family and Identification of Their Functions in Rice Quality and Yield
Source: Int J Mol Sci. 2024 Nov 6;25(22):11931. doi: 10.3390/ijms252211931 (PMC11593806; doi:10.3390/ijms252211931)
Supplement: Supplementary file 1 [file ijms-25-11931-s001.zip › ijms-3255139-supplementary.pdf]

Supplementary Material:

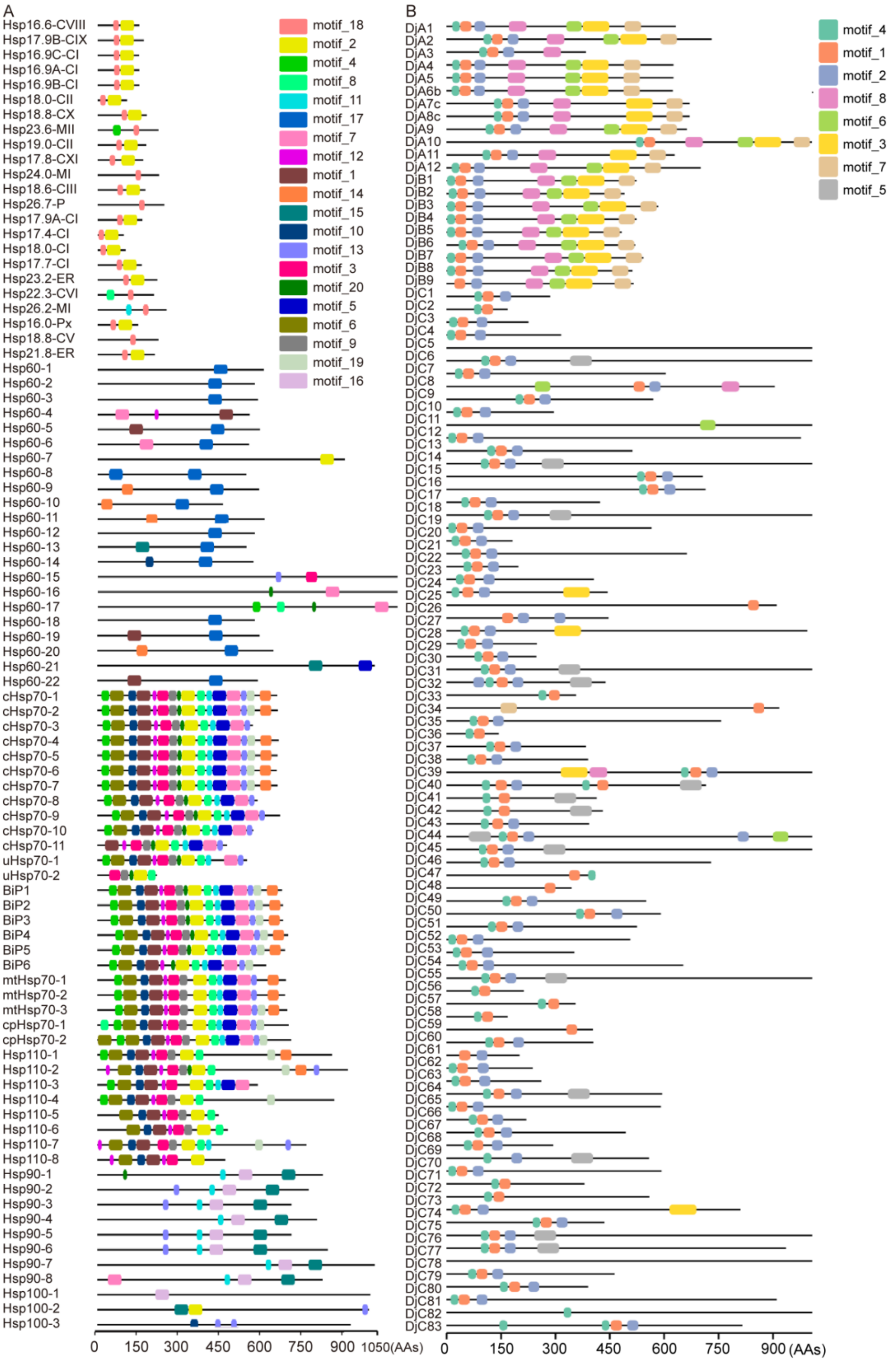

Figure S1. Conserved motifs of the Hsp family genes in rice. (A) Conserved motifs of 23 sHsp, 22 Hsp60, 32 Hsp70 (18 Hsp70, 6 Bips and 8 Hsp110), 8 Hsp90 and 3 Hsp100. (B) Conserved motifs of Hsp40 (DjA-C). The scale at the bottom represents the number of amino acids.

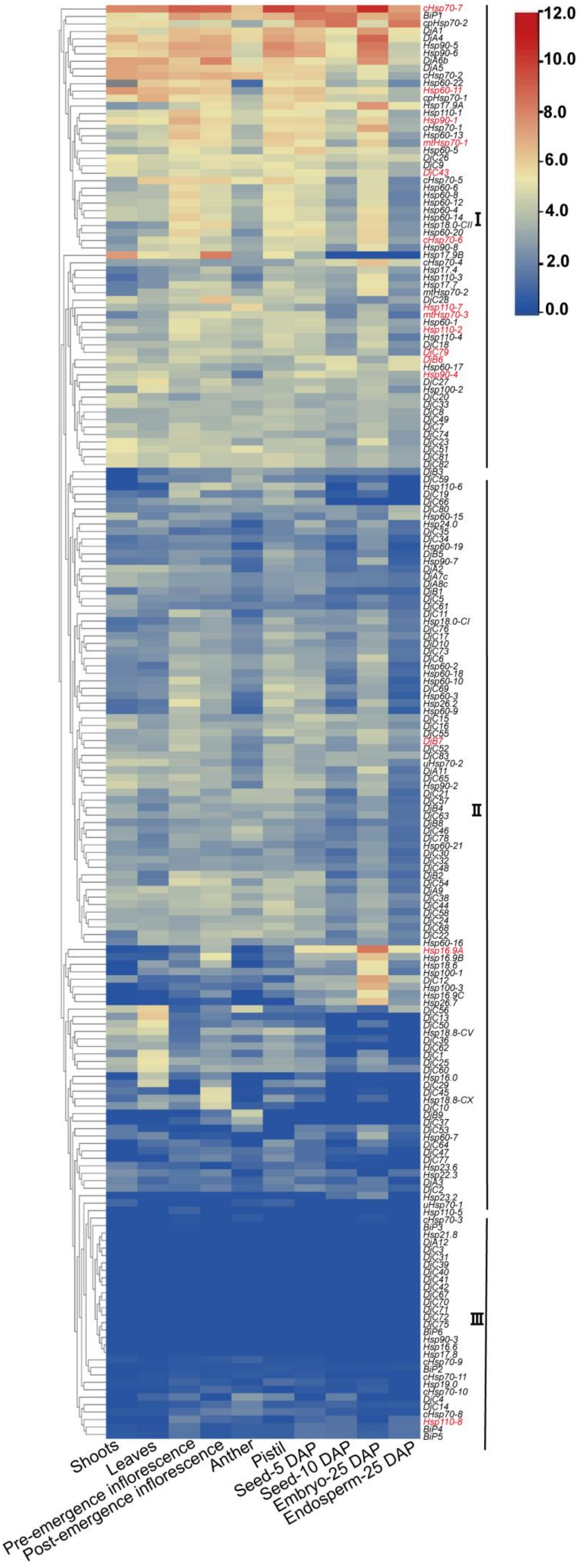
12.0
10.0
8.0
6.0
4.0
2.0
0.0
I
II
III
Shoots
Leaves
Pre-emergence inflorescence
Post-emergence inflorescence
Anther
Pistil
Seed-5 DAP
Seed-10 DAP
Embryo-25 DAP
Endosperm-25 DAP

Figure S2. Expression patterns of 192 Hsps across shoots, leaves, pre-emergence inflorescence, post-emergence inflorescence, anther, pistil, seed, embryo and endosperm. Fifteen genes marked in red were selected for knockout and further investigation in this experiment. The range of blue to red indicate the expression levels from low to high. Clustering according to the expression level in each tissue. The data on expression patterns were retrieved from the RiceData website (https://www.ricedata.cn/gene/index.htm)

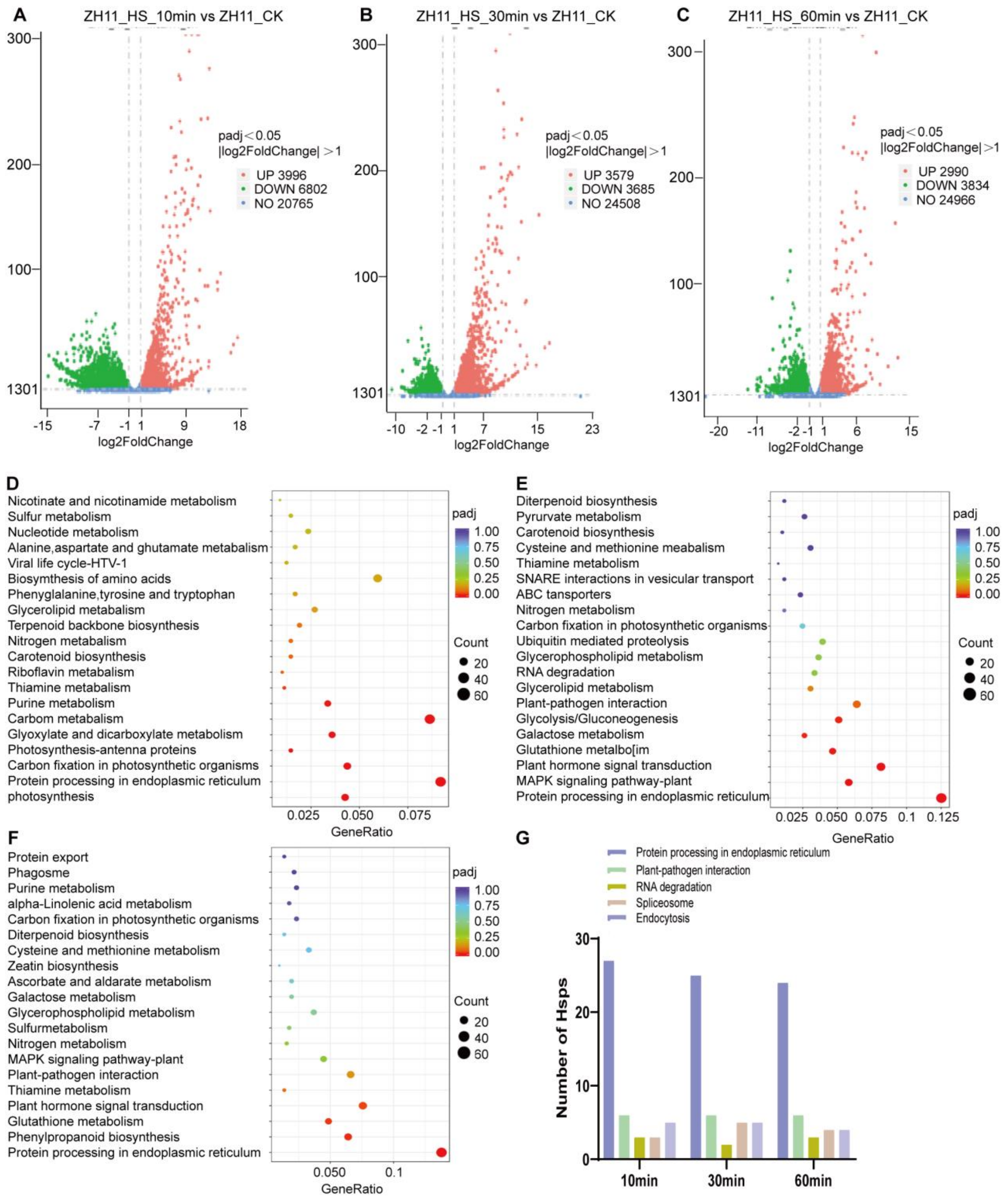

Figure S3. Transcriptome analysis results, including volcano maps and Kyoto Encyclopedia of Genes and Genomes (KEGG) annotations. (A-C) Differentially expressed genes (DEGs) of ZH11_CK and ZH11_HS_10min, ZH11_HS_30min, and ZH11_HS_60min samples. (D-F) KEGG enrichment of significantly up-regulated DEGs in ZH11_HS_10min vs ZH11_CK, ZH11_HS_30min vs ZH11_CK, ZH11_HS_60min vs ZH11_CK. (G) The number of Hsp genes in each function of KEGG annotation under high temperature for 10min, 30min, and 60min. padj indicates the significance level of enrichment and count represents gene numbers.

A

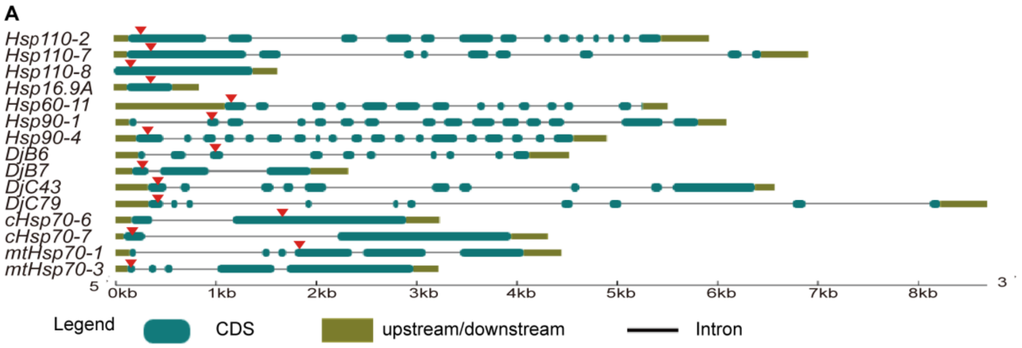

B

	Target PAM	
ZH11	GGTGGACCAGGTGAAGGCCGGACTGGAGA	
hsp16.9a-1	GGTGGACCAGGTGAAGGCCG - ACTGGAGA	-G, 122FS, 142 Premature
hsp16.9a-2	GGTGGACCAGGTGAAGGCCGCGACTGGAGA	+C, 122FS, 141 Premature
ZH11	TGCTTATGAGATCTTGACGGATCAGGAGA	
djb6	TGCTTATGAGATCTTGACGGCATCAGGAGA	+C, 78FS, 86 Premature
ZH11	CGCTGGCGGCCTTGTCGACGCCCAGGATC	
djb7-1	CGCTGGCGGCCTTGTCGAC-CCCAGGATC	-G, 9FS, 28 Premature
djb7-2	CGCTGGCGGC-----------------C	-CTTGTCGACGCCCAGGAT, Deletion (7 ILGVDK)
ZH11	CCTCTGCCGCGCCGCCAACGTCAAGGCCA	
djc43-1	CCTCCGCC-------------------------TCAAGGCCA	T→C and -GCGCCGCCAACG, CRAANV 31 RL
djc43-2	CCTCTGCCGCGCCGCCAACCTCAAGGCCA	G→C, V37L
ZH11	AGTCTCCAACGCCATCTACTGCGAGGAGG	
djc79-1	AGTCTCCAACGCCATCTACTTGCGAGGAGG	+T, 39FS, 42 Premature
djc79-2	AGTCTCCAACGCCATCT --- GCGAGGAGG	-ACT, Deletion (38Y)
ZH11	TTCAACATTCGGTGCCACTTCTACGGTTG	
hsp60-11-1	TTCAACATTCGGTGCCACTT--ACGGTTG	-CT, 9FS, 80 Premature
hsp60-11-2	TTCAACATTCGGTGCCACT---CGGTTG	-TCT, Deletion (9S)
ZH11	CCCAAAGTGATAGAGAATTCAGAAGGCACCAGGACAACAC	
mthsp70-1-1	CCCAAAGTGATAGAGAA-------------------------------CAC	-TTCAGAAGGCACCAGGACAA, 79FS, 85 Premature
mthsp70-1-2	CCCAAAGTGATAGAGAATTCAGCAAGGCACCAGGACAACAC	+A, 81FS, 93 Premature
ZH11	CGGATCTCTCATCGCGTCGAGGCTGGCCA	
mthsp70-3-1	CGGATCTCTCATCGCGTCGAAGGCTGGCCA	+A, 10FS, 81 Premature
mthsp70-3-2	CGGATCTGTCATGGCGTCGAGGCTGGCCA	C→G, C→G Substitution (LI 6 and 7 VM)
ZH11	TGACATGAAGCTATGGCCGTTCAAGGTGG	
chsp70-6-1	TGACATGAAGCTATGGCCGTTTCAAGGTGG	+T, 95FS, 104 Premature
chsp70-6-2	TGACATGAAGCTATGGCC-TTCAAGGTGG	-G, 93FS, 104 Premature
ZH11	CGACCTACTCGTGCGTGGGCGTTTGGCAGCACGACCGCGTGGAGA	
chsp70-7-1	CGACCTACTCGTGCGTGGGCCGTTTGGCAG	+C, 22FS, 72 Premature
chsp70-7-2	CGACCTACTCGTGCGTGG---------------------------------------AGA	-24bp, Deletion (22 GVWQHDRV)
ZH11	TCGCCGCGCTCGCCGACGGCAACCGGCTC	
hsp110-2-1	TCGC-GCGCTCGCCGACGGCAACCGGCTC	-C, 73FS, 186 Premature
hsp110-2-2	TCGCCGCGCTCGCCGACGGCA-CCGGCTC	-A, 78FS, 186 Premature
ZH11	TCGCCATGCTGCTCGGGTACCTCAGGCAG	
hsp110-7-1	TCGCCATGCTGCTCGGGTAC-TCAGGCAG	-C, 128FS, 225 Premature
hsp110-7-2	TCGCCATGCTGCTCGGGTACCCTCAGGCAG	+C, 128FS, 239 Premature
ZH11	ATACCATCAGGTACGGGCCACCGAGGGTG	
hsp110-8-1	ATACCATCA-GTACGGGCCACCGAGGGTG	-G, 32FS, 137 Premature
hsp110-8-2	ATACCATC-GA-ACGGGCCACCGAGGGTG	AGGT→GA, 32FS, 64 Premature
ZH11	GTTAGTTGATCCGCCGAAGGTAGAGGAGA	
hsp90-1-1	GTTAGTTGATCCGC-------------TAGAGGAGA	-CGAAGG, Deletion (42 RR)
hsp90-1-2	GTTAGTTGATCCGCCGAAGGTTAGAGGAGA	+T, 45FS, 60 Premature
ZH11	GCGAGAGGGGTGAGGTGGGAGGCCGGGAG	
hsp90-4	GCGAGAGGGGTGAGGTGGGAAGGCCGGGAG	+A, 52FS, 90 Premature

Figure S4. Target sequence position and mutation mode of the CRISPR/Cas9 generated mutants for 15 genes. (A) The gene structure of the 15 genes. The red arrows point to the position of the knockout target sequence. (B) PAM sequence and changes in amino acids. + indicates the addition of bases, -indicates missing bases and FS represents frame shift.

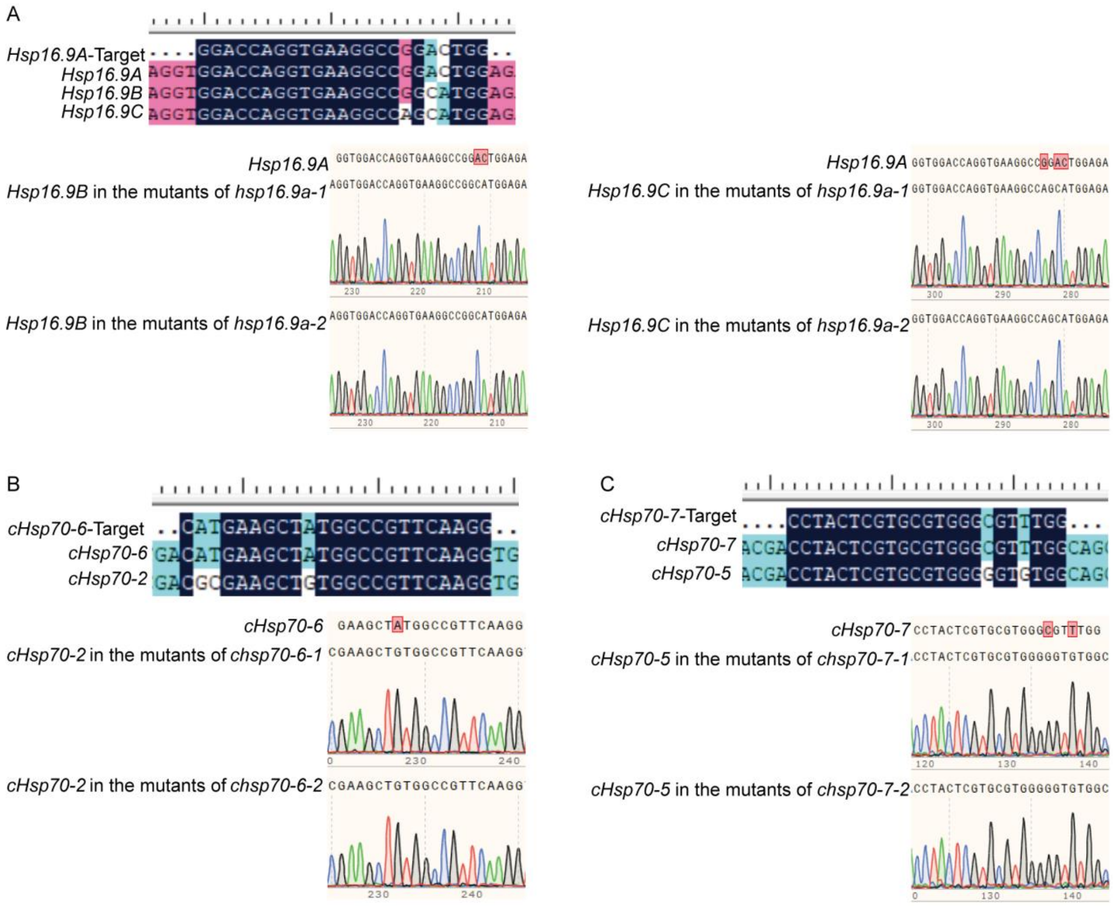

Figure S5. The orthologous genes in the mutants were not knocked out. (A) The homologous genes *Hsp16.9B* and *Hsp16.9C* of *Hsp16.9A* were not knocked out in the *hsp16.9a* mutants. (B) The homologous gene *cHsp70-2 of cHsp70-6* were not knocked out in the *chsp70-6* mutants. (C) The homologous gene *cHsp70-5 of cHsp70-7* were not knocked out in the *chsp70-7* mutants.

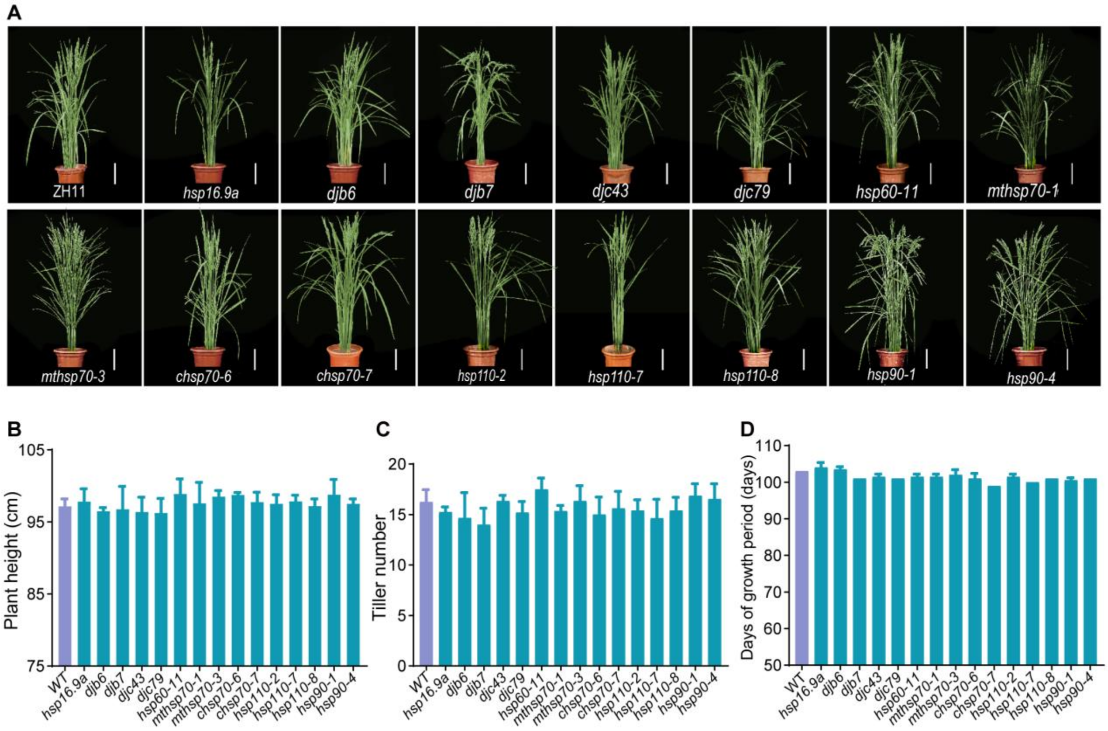

Figure S6. The plant morphology of wild type and CRISPR/Cas9-based mutants. (A) The heading stage plants of ZH11 and the mutants. Scale bar = 20cm. (B) Plant height of ZH11 and the mutants. n=10 (C) Tiller number of ZH11 and the mutants. n=10 (D) Days of growth period of ZH11 and the mutants, grown in Hangzhou on June 12, 2023. Data are means ± SD from at least three biological replicates.

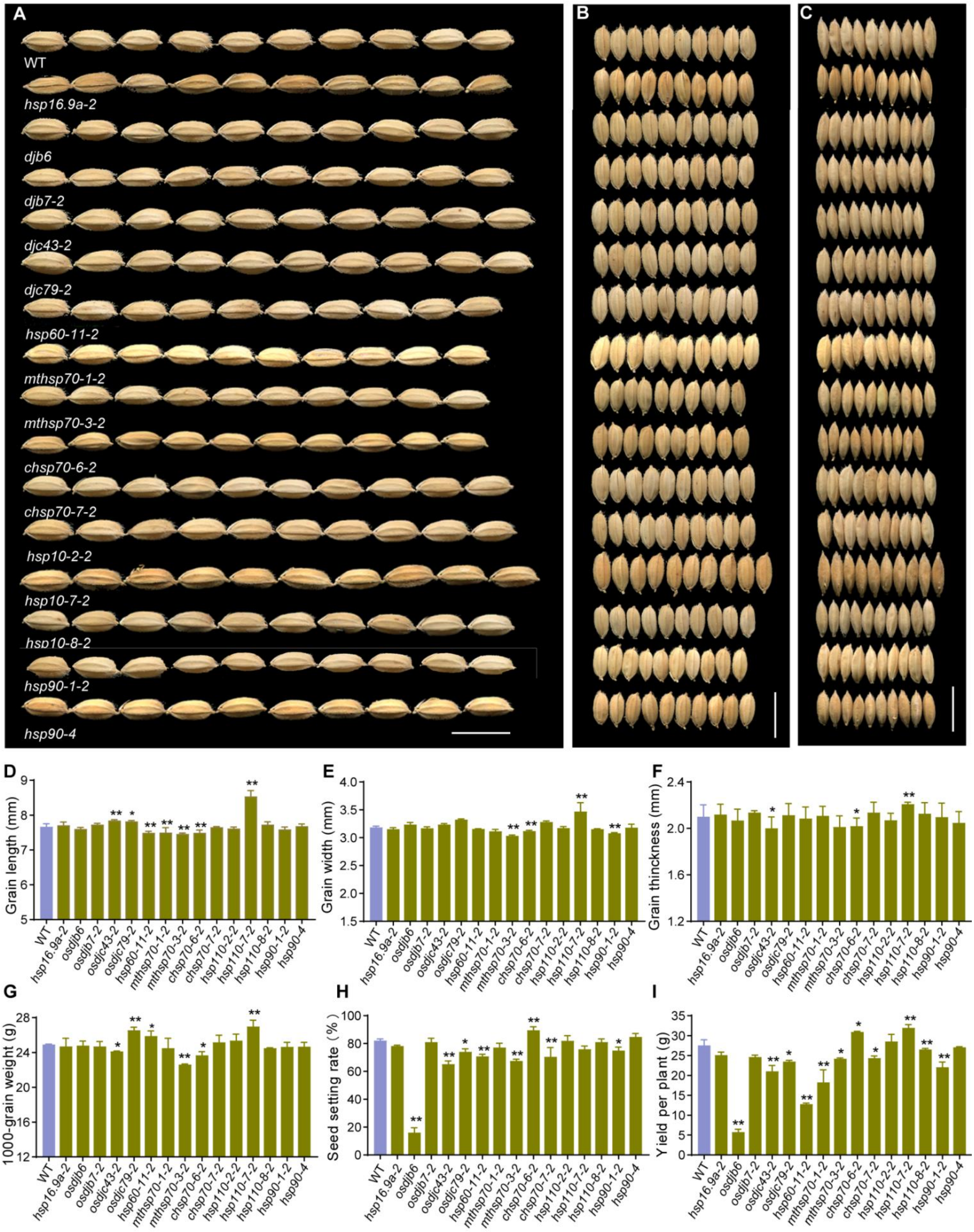

Figure S7. Grain shape and yield related traits of ZH11 and the *hsp* mutants. (A-C) Comparison of the grain length (A), grain width (B), and grain thickness (C) of ZH11 and *hsp* mutants, scale bars=1cm. (D-I) Grain length (D), grain width (E), grain thickness (F), 1000-grain weight (G), seed setting rate (H), and yield per plant (I) of ZH11 and the mutants. The investigated plants were grown in natural high-temperature conditions in the fields of Hangzhou, 2023. Data are means±SD, n=20 in (D-F), n=3 in

(G), and n=10 in (H, I), and no less than 200 grains per replication in (G). Asterisks showed the statistical significance between WT and the mutants, as determined by Student's *t*-test (* $P < 0.05$; ** $P < 0.01$).

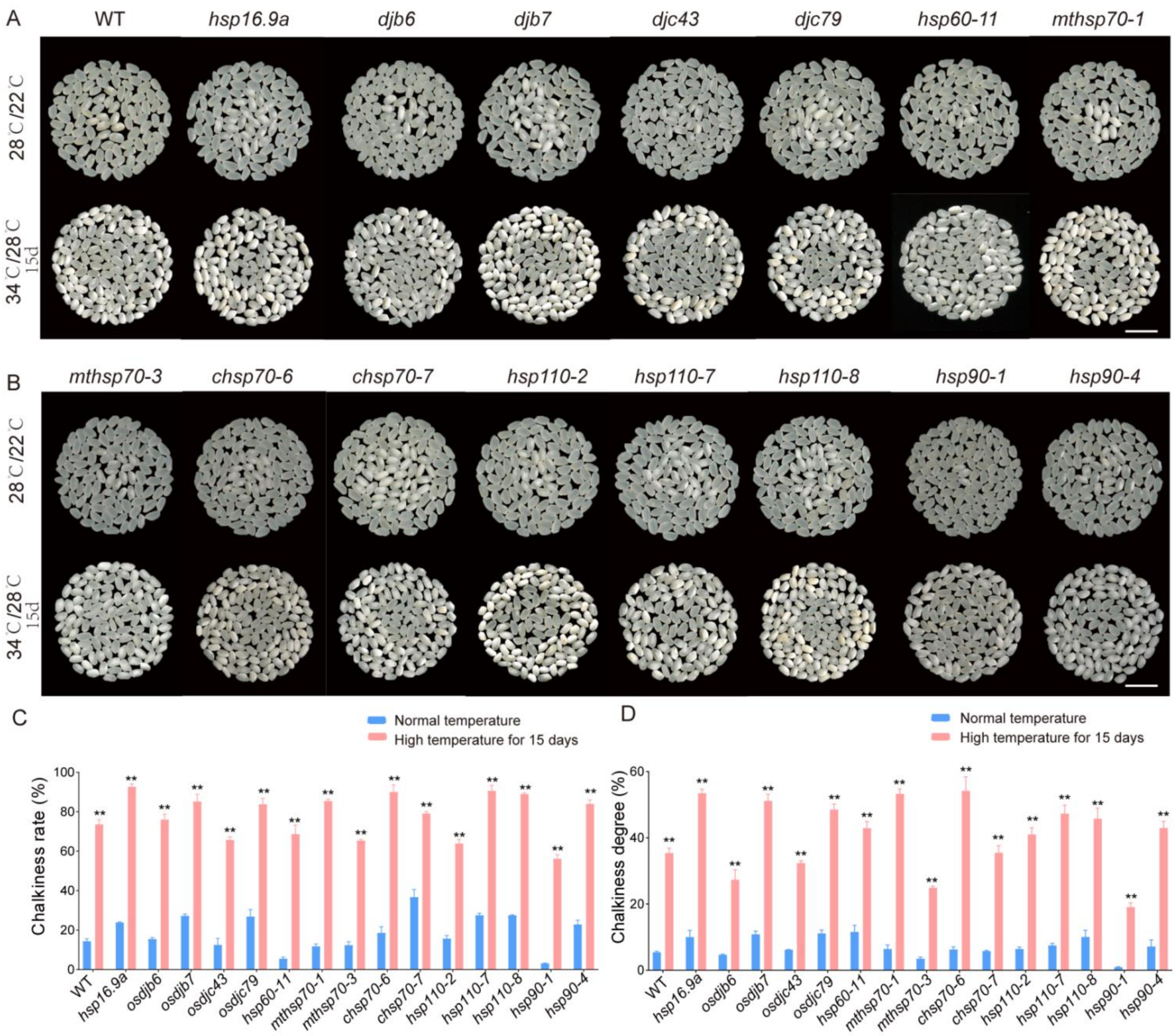

Figure S8. Grain quality of ZH11 and *hsp* mutants under different temperatures. (A-B) Appearance of mature grains of ZH11 and 15 *hsp* mutants at normal temperature (22℃/28℃) and high temperature (28℃/34℃) for 15 days. Scale bars = 1cm. (C-D) Chalkiness rate and chalkiness degree of ZH11 and 15 *hsp* mutants at normal temperature and high temperature. Data are means ± SD (n=3), and no less than 200 grains per replication in (B, C). Asterisks showed the statistical significance between WT and the mutants, as determined by Student's *t*-test (* $P < 0.05$; ** $P < 0.01$).

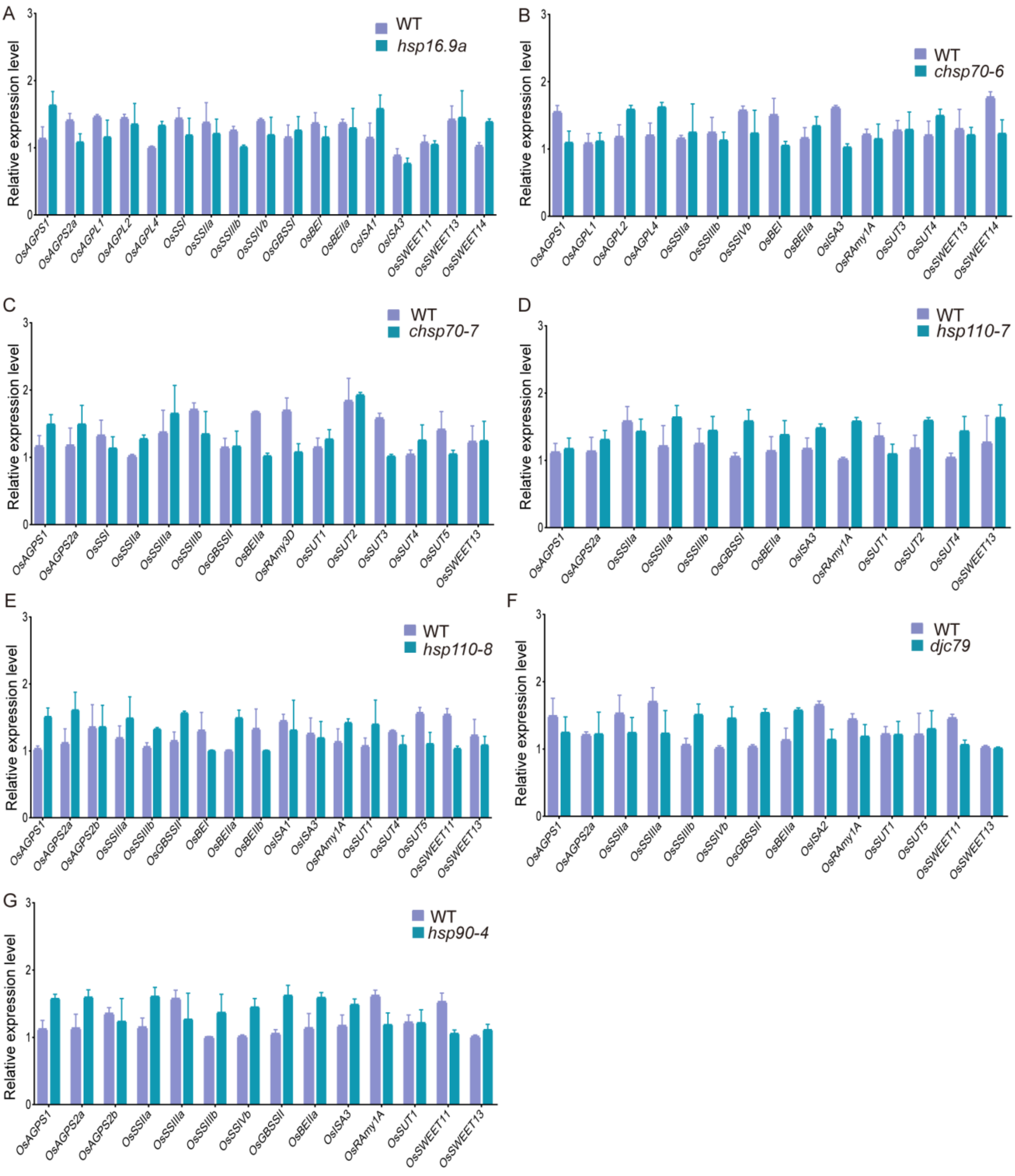

Figure S9. The expression levels of starch synthesis-related genes in developing endosperm of wild-type (WT) and 7 mutants (*hsp16.9a*, *chsp70-6*, *chsp70-7*, *hsp110-7*, *hsp110-8*, *djc79*, *and hsp90-4*). (A-G) The relative expression level of starch synthesis-related genes in 10d endosperm after flowering under nature high temperature in Hangzhou field in 2023. The data presented here are the relative expression levels of the genes that are expressed differently between mutation and wild-type. The rice *UBIQUITIN* gene was used as the internal control. Data are means ± SD of three individual replicates.

Table S1. The 192 Hsp genes were analyzed in this study.

	**Original_ID**	**MSU**	**RAP**
1	*Hsp16.6-CVIII*	LOC_Os01g04340	Os01g0135800
2	*Hsp17.9B-CIX*	LOC_Os01g04350	Os01g0135900
3	*Hsp16.9C-CI*	LOC_Os01g04360	Os01g0136000
4	*Hsp16.9A-CI*	LOC_Os01g04370	Os01g0136100
5	*Hsp16.9B-CI*	LOC_Os01g04380	Os01g0136200
6	*Hsp18.0-CII*	LOC_Os01g08860	Os01g0184100
7	*Hsp18.8-CX*	LOC_Os02g03570	Os02g0128000
8	*Hsp23.6-MII*	LOC_Os02g10710	Os02g0201000
9	*Hsp19.0-CII*	LOC_Os02g12610	Os02g0217850
10	*Hsp17.8-CXI*	LOC_Os02g48140	Os02g0711300
11	*Hsp24.0-MI*	LOC_Os02g52150	Os02g0758000
12	*Hsp18.6-CIII*	LOC_Os02g54140	Os02g0782500
13	*Hsp26.7-P*	LOC_Os03g14180	Os03g0245800
14	*Hsp17.9A-CI*	LOC_Os03g15960	Os03g0266300
15	*Hsp17.4-CI*	LOC_Os03g16020	Os03g0266900
16	*Hsp18.0-CI*	LOC_Os03g16030	Os03g0267000
17	*Hsp17.7-CI*	LOC_Os03g16040	Os03g0267200
18	*Hsp23.2-ER*	LOC_Os04g36750	Os04g0445100
19	*Hsp22.3-CVI*	LOC_Os05g42120	Os05g0500500
20	*Hsp26.2-MI*	LOC_Os06g11610	Os06g0219500
21	*Hsp16.0-Px*	LOC_Os06g14240	Os06g0253100
22	*Hsp18.8-CV*	LOC_Os07g33350	Os07g0517100
23	*Hsp21.8-ER*	LOC_Os11g13980	Os11g0244200
24	*DjA1*	LOC_Os02g43930	Os02g0656500
25	*DjA2*	LOC_Os02g56040	Os02g0804500
26	*DjA3*	LOC_Os03g12236	Os03g0222800
27	*DjA4*	LOC_Os03g44620	Os03g0648400
28	*DjA5*	LOC_Os03g57340	Os03g0787300
29	*DjA6b*	LOC_Os04g46390	Os04g0549600
30	*DjA7c*	LOC_Os05g26902	Os05g0333600
31	*DjA8c*	LOC_Os05g26926	Os05g0334400
32	*DjA9*	LOC_Os06g02620	Os06g0116800
33	*DjA10*	LOC_Os06g11440	Os06g0218150
34	*DjA11*	LOC_Os12g07060	Os12g0168400
35	*DjA12*	LOC_Os12g42440	Os12g0619100
36	*DjB1*	LOC_Os01g13760	Os01g0239100
37	*DjB2*	LOC_Os01g65480	Os01g0875700
38	*DjB3*	LOC_Os02g03600	Os02g0128400
39	*DjB4*	LOC_Os02g20394	Os02g0306900
40	*DjB5*	LOC_Os05g03630	Os05g0127400
41	*DjB6*	LOC_Os05g06440	Os05g0156500
42	*DjB7*	LOC_Os05g48810	Os05g0562300

43	*DjB8*	LOC_Os08g06460	Os08g0161900
44	*DjB9*	LOC_Os08g28700	Os08g0374400
45	*DjC1*	LOC_Os01g01160	Os01g0101700
46	*DjC2*	LOC_Os01g06454	Os01g0157800
47	*DjC3*	LOC_Os01g17030	
48	*DjC4*	LOC_Os01g17040	
49	*DjC5*	LOC_Os01g25320	Os01g0355500
50	*DjC6*	LOC_Os01g27740	Os01g0375100
51	*DjC7*	LOC_Os01g32870	Os01g0512100
52	*DjC8*	LOC_Os01g33800	Os01g0521500
53	*DjC9*	LOC_Os01g37560	Os01g0556400
54	*DjC10*	LOC_Os01g42190	Os01g0606900
55	*DjC11*	LOC_Os01g44310	Os01g0634300
56	*DjC12*	LOC_Os01g50700	OsO1g0702450
57	*DjC13*	LOC_Os01g53020	Os01g0730500
58	*DjC14*	LOC_Os01g69930	Os01g0923800
59	*DjC15*	LOC_Os01g74580	Os01g0977200
60	*DjC16*	LOC_Os02g10180	Os02g0195300
61	*DjC17*	LOC_Os02g10220	Os02g0195800
62	*DjC18*	LOC_Os02g30620	Os02g0510000
63	*DjC19*	LOC_Os02g35000	Os02g0555700
64	*DjC20*	LOC_Os02g46640	Os02g0693200
65	*DjC21*	LOC_Os02g50760	Os02g0741100
66	*DjC22*	LOC_Os02g52270	Os02g0760000
67	*DjC23*	LOC_Os02g54130	Os02g0782300
68	*DjC24*	LOC_Os03g04400	Os03g0136800
69	*DjC25*	LOC_Os03g10180	Os03g0198300
70	*DjC26*	LOC_Os03g15480	Os03g0261500
71	*DjC27*	LOC_Os03g18200	Os03g0293000
72	*DjC28*	LOC_Os03g18870	Os03g0300600
73	*DjC29*	LOC_Os03g20730	Os03g0323600
74	*DjC30*	LOC_Os03g28310	Os03g0401200
75	*DjC31*	LOC_Os03g36160	Os03g0560400
76	*DjC32*	LOC_Os03g51830	Os03g0728100
77	*DjC33*	LOC_Os03g54150	Os03g0752700
78	*DjC34*	LOC_Os03g55360	Os03g0761700
79	*DjC35*	LOC_Os03g56540	Os03g0776900
80	*DjC36*	LOC_Os03g60790	Os03g0822800
81	*DjC37*	LOC_Os03g61550	Os03g0831000
82	*DjC38*	LOC_Os03g61730	Os03g0832900
83	*DjC39*	LOC_Os03g62120	Os03g0837400
84	*DjC40*	LOC_Os03g62130	Os03g0837550
85	*DjC41*	LOC_Os03g62140	
86	*DjC42*	LOC_Os03g62150	Os03g0837700

87	*DjC43*	LOC_Os04g24180	Os04g0307200
88	*DjC44*	LOC_Os04g31940	Os04g0388800
89	*DjC45*	LOC_Os04g57880	Os04g0675400
90	*DjC46*	LOC_Os04g59060	Os04g0687300
91	*DjC47*	LOC_Os05g01590	Os05g0106500
92	*DjC48*	LOC_Os05g30130	Os05g0364500
93	*DjC49*	LOC_Os05g31062	Os05g0374600
94	*DjC50*	LOC_Os05g45350	Os05g0529700
95	*DjC51*	LOC_Os05g46620	Os05g0543700
96	*DjC52*	LOC_Os05g50370	Os05g0579900
97	*DjC53*	LOC_Os06g09560	Os06g0195800
98	*DjC54*	LOC_Os06g13060	Os06g0237800
99	*DjC55*	LOC_Os06g34440	Os06g0535300
100	*DjC56*	LOC_Os06g44160	Os06g0650900
101	*DjC57*	LOC_Os07g03270	Os07g0124800
102	*DjC58*	LOC_Os07g09450	Os07g0192300
103	*DjC59*	LOC_Os07g28800	Os07g0470800
104	*DjC60*	LOC_Os07g43330	Os07g0626400
105	*DjC61*	LOC_Os07g44310	Os07g0637100
106	*DjC62*	LOC_Os08g35160	Os08g0452900
107	*DjC63*	LOC_Os08g36980	Os08g0474600
108	*DjC64*	LOC_Os08g37270	
109	*DjC65*	LOC_Os08g41110	Os08g0522600
110	*DjC66*	LOC_Os08g43490	Os08g0548400
111	*DjC67*	LOC_Os09g20320	Os09g0368800
112	*DjC68*	LOC_Os09g28590	Os09g0460000
113	*DjC69*	LOC_Os09g28890	Os09g0463700
114	*DjC70*	LOC_Os09g32050	Os09g0493800
115	*DjC71*	LOC_Os10g03610	Os10g0124600
116	*DjC72*	LOC_Os10g11012	Os10g0188200
117	*DjC73*	LOC_Os10g36370	Os10g0507800
118	*DjC74*	LOC_Os10g42439	Os10g0575200
119	*DjC75*	LOC_Os11g36530	Os11g0573800
120	*DjC76*	LOC_Os11g36960	Os11g0578100
121	*DjC77*	LOC_Os11g37000	Os11g0578500
122	*DjC78*	LOC_Os11g43950	Os11g0661200
123	*DjC79*	LOC_Os12g15590	Os12g0258200
124	*DjC80*	LOC_Os12g27070	Os12g0456200
125	*DjC81*	LOC_Os12g31840	Os12g0502700
126	*DjC82*	LOC_Os12g36180	Os12g0548200
127	*DjC83*	LOC_Os12g41820	Os12g0612400
128	*Hsp60-1*	LOC_Os02g01280	Os02g0102900
129	*Hsp60-2*	LOC_Os02g14929	Os02g0247200
130	*Hsp60-3*	LOC_Os03g04970	Os03g0143400

131	*Hsp60-4*	LOC_Os03g59020	Os03g0804800
132	*Hsp60-5*	LOC_Os03g64210	Os03g0859600
133	*Hsp60-6*	LOC_Os04g46620	Os04g0551800
134	*Hsp60-7*	LOC_Os04g59540	Os04g0691900
135	*Hsp60-8*	LOC_Os05g05470	Os05g0147400
136	*Hsp60-9*	LOC_Os05g46290	Os05g0540300
137	*Hsp60-10*	LOC_Os05g48290	Os05g0556700
138	*Hsp60-11*	LOC_Os06g02380	Os06g0114000
139	*Hsp60-12*	LOC_Os06g34690	Os06g0538000
140	*Hsp60-13*	LOC_Os06g36700	Os06g0562600
141	*Hsp60-14*	LOC_Os06g47320	Os06g0687700
142	*Hsp60-15*	LOC_Os08g01390	Os08g0104700
143	*Hsp60-16*	LOC_Os08g33200	Os08g0428900
144	*Hsp60-17*	LOC_Os08g34950	Os08g0450800
145	*Hsp60-18*	LOC_Os09g11250	Os09g0284400
146	*Hsp60-19*	LOC_Os09g38980	Os09g0563300
147	*Hsp60-20*	LOC_Os10g32550	Os10g0462900
148	*Hsp60-21*	LOC_Os12g13440	Os12g0236700
149	*Hsp60-22*	LOC_Os12g17910	Os12g0277500
150	*cHsp70-1*	LOC_Os01g62290	Os01g0840100
151	*cHsp70-2*	LOC_Os03g16860	Os03g0276500
152	*cHsp70-3*	LOC_Os03g16880	Os03g0276800
153	*cHsp70-4*	LOC_Os03g16920	Os03g0277300
154	*cHsp70-5*	LOC_Os03g60620	Os03g0821100
155	*cHsp70-6*	LOC_Os05g38530	Os05g0460000
156	*cHsp70-7*	LOC_Os11g47760	Os11g0703900
157	*cHsp70-8*	LOC_Os11g08440	Os11g0187500
158	*cHsp70-9*	LOC_Os11g08445	
159	*cHsp70-10*	LOC_Os11g08460	Os11g0187600
160	*cHsp70-11*	LOC_Os11g08470	Os11g0187800
161	*uHsp70-1*	LOC_Os01g49430	OsO100688900
162	*uHsp70-2*	LOC_Os12g38180	Os12g0569700
163	*BiP1*	LOC_Os02g02410	Os02g0115900
164	*BiP2*	LOC_Os03g50250	Os03g0710500
165	*BiP3*	LOC_Os05g30480	Os05g0367800
166	*BiP4*	LOC_Os05g35400	Os05g0428600
167	*BiP5*	LOC_Os08g09770	Os08g0197700
168	*BiP6*	LOC_Os01g33360	Os01g0517850
169	*mtHsp70-1*	LOC_Os02g53420	Os02g0774300
170	*mtHsp70-2*	LOC_Os03g02260	Os03g0113700
171	*mtHsp70-3*	LOC_Os09g31486	Os09g0491772
172	*cpHsp70-1*	LOC_Os05g23740	Os05g0303000
173	*cpHsp70-2*	LOC_Os12g14070	Os12g0244100
174	*Hsp110-1*	LOC_Os01g08560	Os01g0180800

175	*Hsp110-2*	LOC_Os02g48110	Os02g0710900
176	*Hsp110-3*	LOC_Os03g11910	Os03g0218500
177	*Hsp110-4*	LOC_Os05g08840	Os05g0181000
178	*Hsp110-5*	LOC_Os05g51360	Os05g0591400
179	*Hsp110-6*	LOC_Os06g10990	Os06g0212900
180	*Hsp110-7*	LOC_Os06g46600	Os06g0679800
181	*Hsp110-8*	LOC_Os12g05760	
182	*Hsp90-1*	LOC_Os06g50300	Os06g0716700
183	*Hsp90-2*	LOC_Os08g38086	Os08g0487800
184	*Hsp90-3*	LOC_Os08g39140	Os08g0500700
185	*Hsp90-4*	LOC_Os09g29840	Os09g0474300
186	*Hsp90-5*	LOC_Os09g30412	Os09g0482100
187	*Hsp90-6*	LOC_Os09g30418	Os09g0482600
188	*Hsp90-7*	LOC_Os09g36420	Os09g0534600
189	*Hsp90-8*	LOC_Os12g32986	Os12g0514500
190	*Hsp100-1*	LOC_Os02g08490	Os02g0181900
191	*Hsp100-2*	LOC_Os03g31300	Os03g0426900
192	*Hsp100-3*	LOC_Os05g44340	Os05g0519700

Table S2. The data of chalkiness rate and chalkiness degree at different temperatures.

	The chalkiness rate of seeds （%）			The chalkiness degree of seeds （%）		
	Normal temperature	High temperature	Difference between high and normal temperatures	Normal temperature	High temperature	Difference between high and normal temperatures
WT	14.35±1.23	68.63±2.32	54.28	5.43±1.01	35.43±1.47	30
hsp16.9a	**23.89±0.28**	**87.67±1.47**	**63.78****	**10.04±0.09**	**53.44±1.25**	**43.4****
djb6	**15.00±0.98**	**71.88±1.44**	**56.88**	5.80±1.83	35.5±2.15	29.7
djb7	27.25±1.04	80.22±3.56	52.97	**10.87±1.02**	**51.14±3.44**	**40.27****
djc43	12.51±2.31	60.66±1.66	48.15	6.19±0.92	32.35±0.73	26.16
djc79	26.84±3.57	78.87±2.92	52.03	**11.13±1.00**	**48.55±1.64**	**37.42****
hsp60-11	**5.01±1.31**	**63.77±1.31**	**58.76***	4.62±0.78	27.49±2.15	22.87
mthsp70-1	**11.88±2.10**	**80.51±0.91**	**68.63****	**6.55±0.93**	**53.26±1.50**	**46.71****
mthsp70-3	12.34±1.80	60.82±2.74	48.48	3.51±0.48	25.17±2.68	21.66
chsp70-6	**19.62±3.10**	**85.07±3.58**	**65.45****	**6.26±0.82**	**54.17±4.23**	**47.91****
chsp70-7	36.75±3.80	77.42±1.39	40.67	**11.57±1.25**	**42.91±1.79**	**31.34**
hsp110-2	15.76±1.50	68.88±2.13	53.12	**6.42±0.56**	**41.00±2.50**	**34.58***
hsp110-7	**27.55±1.05**	**85.65±2.74**	**58.1***	**12.52±0.64**	**47.28±2.61**	**34.76***
hsp110-8	**27.59±1.09**	**83.99± 0.49**	**56.4**	**10.07±2.09**	**45.76±3.22**	**35.69****
hsp90-1	**3.13±0.52**	**66.21±1.98**	**63.08****	0.91±0.58	19.10±1.29	18.19
hsp90-4	**22.92±0.32**	**79.04±1.98**	**56.12**	7.14±0.84	43.30±2.21	**36.16****

The data marked in red indicated that the difference of chalkiness rate or degree under different temperatures was greater than WT. Asterisks showed the statistical significance between WT and the mutants, as determined by Student's *t*-test (* $p < 0.05$; ** $p < 0.01$).

Table S3. Primers used in this study.

Primer name	Sequence
CR-Hsp16.9A-F	TAGAGCAACCATGTCGCTGG
CR-Hsp16.9A-R	CACACCAGCAGCAGACCATA
CR-DjB6-F	CCCCGACAAGAATCCCAACA
CR-DjB6-R	CACATTGGCCTCACCTGCTA
CR-DjB7-F	GAATAGCCAAGAACGCGACG
CR-DjB7-R	CTGCTTACCTCGTACGCCTC
CR-DjC43-F	GACTGTTTGTTGGTGGTGCG
CR-DjC43-R	AGGAGCGAATCCCCCAGTAT
CR-DjC79-R	GATCTCCGATACGTTCGCGT
CR-DjC79-F	CTCTCCCGTCTCCGTATCCT
CR-Hsp60-5-F	CCTCCACCTCAGCTTCCATTT
CR-Hsp60-5-R	CTTGATAGCCGAGCCATCCT
CR-mtHsp70-1-F	TTTCAGTTGACCGCCAATGC
CR-mtHsp70-1-R	TTGTGGGTTGGTCACTGCTT
CR-mtHsp70-3-F	GCGAGGGGTTTAAGAGCGAA
CR-mtHsp70-3-R	GAGACACACGAGTTGGTCGT
CR-cHsp70-6-F	AGTCTCACTGCTGTAGTGCTG
CR-cHsp70-6-R	CCTCTGCGAGTCGTTGAAGT
CR-cHsp70-7-F	CTTGGTTCCAATTCGTCGCC
CR-cHsp70-7-R	GGTGCTCACGTAGGTGTTGA
CR-Hsp110-2-F	GGTGGCCATCAACGAGATGT
CR-Hsp110-2-R	AGGACGTTAACTCCGGCAAG
CR-Hsp110-7-F	CTCCTCAACGCCGAGTCCAA
CR-Hsp110-7-R	CCTGCGTGAAGTAGCAAGGG
CR-Hsp110-8-F	GCTATGCTGTTTTCATCTGCCG
CR-Hsp110-8-R	GTAGTAGGGGACGGTGACGA
CR-Hsp90-1-F	ATCTGCTATTGCGCACCGTA
CR-Hsp90-1-R	CCTCCCTGCACACATTCCAT
CR-Hsp90-4-F	GCACAGAGGTATAGTGGTGGG
CR-Hsp90-4-R	ACATTCGACCTAGACGCGGA
1132-Hsp16.9A-F	CGGGCTGCAGGAATTCCCCATTCTCCCTCGACCTCT
1132-Hsp16.9A-R	CGGTATCGATAAGCTTACCGGAGATCTCAATGGCCT
1132-OsDjB6-F	CGGGCTGCAGGAATTCATGGCGGCGCCGCGGTGGAT
1132-OsDjB6-R	CGGTATCGATAAGCTTTGTGAGAATGCTCTTCAGCT
1132-OsDjB7-F	TCCCCCGGGCTGCAGGAATTCATGGGGATGGATTACTACAA
1132-OsDjB7-R	TCGACCGGTATCGATAAGCTTCTGCCCCAGCAGCCGCTTGA
1132-OsDjC43-F	CGGGCTGCAGGAATTCATGGCGGCGGCGGAGGAGAA
1132-OsDjC43-R	CGGTATCGATAAGCTTATCATCACTGCCAGAATCTA
1132-OsDjC79-F	CGGGCTGCAGGAATTCATGTCCCAAGTTGGATCCGC
1132-OsDjC79-R	CGGTATCGATAAGCTTTCTTCTACGCTTTGATTCCT
1132-Hsp60-5-F	CGGGCTGCAGGAATTCATGGCTTCAACATTCGGTGC
1132-Hsp60-5-R	CGGTATCGATAAGCTTGTACCCGTAGCCGGAGTTGT

1132-mtHsp70-1-F	CGGGCTGCAGGAATTCATGGCGGCGTCGCTGCTTCT
1132-mtHsp70-1-R	CGGTATCGATAAGCTTCTTCTTGACCTCCTCGTACT
1132-mtHsp70-3-F	CGGGCTGCAGGAATTCATGGCCATCGGATCTCTCAT
1132-mtHsp70-3-R	CGGTATCGATAAGCTTCATCTTAGCTTCCTTAGCAG
1132-cHsp70-6-F	CGGGCTGCAGGAATTCATGTCGAAGGGGGAAGGGCC
1132-cHsp70-6-R	CGGTATCGATAAGCTTGTCGACCTCCTCGATCTTGG
1132-cHsp70-7-F	TCCCCCGGGCTGCAGGAATTCATGGCGGGCAAGGGCGAGGG
1132-cHsp70-7-R	TCGACCGGTATCGATAAGCTTGTCGACCTCCTCGATCTTGG
1132-Hsp110-2-F	CGGGCTGCAGGAATTCATGGCGCCGCCCCGCACATC
1132-Hsp110-2-R	CGGTATCGATAAGCTTCAACTCATCATGAGCTTCAG
1132-Hsp110-7-F	CGGGCTGCAGGAATTCATGAGCGTGGTGGGCTTCGA
1132-Hsp110-7-R	CGGTATCGATAAGCTTGTCTCTATCTGTTGTATGCA
1132-Hsp110-8-F	CGGGCTGCAGGAATTCATGGCTCGCCACATCTTGGC
1132-Hsp110-8-R	CGGTATCGATAAGCTTAAATTCATGGTAACAGCGAT
1132-Hsp90-1-F	CGGGCTGCAGGAATTCATGCGCAAGTGGGCGCTCTC
1132-Hsp90-1-R	CGGTATCGATAAGCTTCAGCTCGTCCTTATCATACG
1132-Hsp90-4-F	CGGGCTGCAGGAATTCATGGCGCCGGCGCTGAGCAG
1132-Hsp90-4-R	CGGTATCGATAAGCTTATCCTTCCATGGATCACTCT
RT-OsAGPS1-F	GTGCCACTTAAAGGCACCATT
RT-OsAGPS1-R	CCCACATTTCAGACACGGTTT
RT-OsAGPS2a-F	ACTCCAAGAGCTCGCAGACC
RT-OsAGPS2a-R	GCCTGTAGTTGGCACCCAGA
RT-OsAGPS2b-F	AACAATCGAAGCGCGAGAAA
RT-OsAGPS2b-R	GCCTGTAGTTGGCACCCAGA
RT-OsAGPL1-F	GGAAGACGGATGATCGAGAAAG
RT-OsAGPL1-R	CACATGAGATGCACCAACGA
RT-OsAGPL2-F	AGTTCGATTCAAGACGGATAGC
RT-OsAGPL2-R	CGACTTCCACAGGCAGCTTATT
RT-OsSSI-F	GGGCCTTCATGGATCAACC
RT-OsSSI-R	CCGCTTCAAGCATCCTCATC
RT-OsSSIIa-F	GCCAATGCCAGGAAGATGA
RT-OsSSIIa-R	GCGCAACATAGGATGGGTTT
RT-OsSSⅢa-F	GCCTGCCCTGGACTACATTG
RT-OsSSⅢa-R	GCAAACATATGTACACGGTTCTGG
RT-OsSSⅢb-F	GTGTGGATTACGCCCTTGAC
RT-OsSSⅢb-R	AGGTCGGTTCCATGTCCAAT
RT-OsSSⅣb-F	GAGCTGCTCCTGCTCAAGAT
RT-OsSSⅣb-R	ACACAATTGCACCCTTGACA
RT-OsBEI-F	TGGCCATGGAAGAGTTGGC
RT-OsBEI-R	CAGAAGCAACTGCTCCACC
RT-OsBEIIa-F	GCCAATGCCAGGAAGATGA
RT-OsBEIIa-R	GCGCAACATAGGATGGGTTT
RT-OsBEIIb-F	ATGCTAGAGTTTGACCGC
RT-OsBEIIb-R	AGTGTGATGGATCCTGCC

RT-OsISA1-F	TGCTCAGCTACTCCTCCATCATC
RT-OsISA1-R	AGGACCGCACAACTTCAACATA
RT-OsISA2-F	ATGCCAATGCCGTTTCTCTC
RT-OsISA2-R	GTGGATGTACGGATCGAGGT
RT-OsISA3-F	ACAGCTTGAGACACTGGGTTGAG
RT-OsISA3-R	GCATCAAGAGGACAACCATCTG
RT-OsGBSSI-F	AACGTGGCTGCTCCTTGAA
RT-OsGBSSI-R	TTGGCAATAAGCCACACACA
RT-OsGBSSII-F	AGCCATGTGGTCTCATCCAA
RT-OsGBSSII-R	ACGTCCACTGGATCAACAGT
RT-OsBIP1-F	TGGAAAGCTGAGGAGGGAAG
RT-OsBIP1-R	CTTGACAGGTCCCATGGTCT
RT-RAmy1A-F	GGGTCTCAAGGAGGAGATCG
RT-RAmy1A-R	CGAGGTAGAGATCGCTGTCA
RT-RAmy3D-F	CAAGGGATACTCCACGGACA
RT-RAmy3D-R	CCTTGGTGGTGAAGTCGAAC
RT-OsSUT1-F	TCATCCCTCAGGTGGTCATCG
RT-OsSUT1-R	CTTGGAGATCTTGGGCAGCAG
RT-OsSUT2-F	GGCGGCCTAGCGGCGGCG
RT-OsSUT2-R	CGAAGGCGTGCGGGATGC
RT-OsSUT3-F	CCGTCGACATGGAGCTCGA
RT-OsSUT3-R	AACGTACGGGGTGAGGAGA
RT-OsSUT4-F	CGCCGGCGGTGGCGGCCTCA
RT-OsSUT4-R	CGTGAGGAGCGAGAGCTGA
RT-OsSUT5-F	CTAGTGCGAAACTCCATCAAA
RT-OsSUT5-R	AAAATATTTGGGTTTCCTGAGAT
RT-OsSWEET11-F	GACGTTCTTG CAGGTGTACA
RT-OsSWEET11-R	TAGCGGACGATGTAGGCGGC
RT-OsSWEET13-F	GCCTCCTTGGCAACCTCATA
RT-OsSWEET13-R	TACGGCACCGACTGGAACCC
RT-OsSWEET14-F	TTCCCAACGTGCTGGGCTTCT
RT-OsSWEET14-R	GCACCTCGCGGGTCTTGACG
